# A masking effect of vitamin D on ASD symptom severity: mediation by developmental level and altered predictive features in vitamin D insufficiency

**DOI:** 10.3389/fpsyt.2026.1900932

**Published:** 2026-08-28

**Authors:** Shimei Lu, Liangqiong Deng, Xinyi Jia, Xiya Lang, Lianghui Diao, Yafei Wang, Pingjing Wen

**Affiliations:** 1 Department of Preventive Medicine, Guangxi University of Chinese Medicine, Nanning, China; 2 Department of Children Healthcare, Liuzhou Hospital of Guangzhou Women and Children’s Medical Center, Liuzhou, Guangxi, China

**Keywords:** ASD, developmental level, machine learning, masking effect, vitamin D

## Abstract

**Background:**

Symptom severity in children with autism spectrum disorder (ASD) correlates with developmental level. Vitamin D is crucial to developmental level. Studies show inconsistent findings on serum 25(OH)D and ASD symptom severity. This study explores their association and the mediating role of developmental level.

**Methods:**

A total of 699 children aged 24–71 months were enrolled. Serum 25(OH)D levels were measured and children were categorized into normal vitamin D (NVD) and vitamin D insufficiency (VDI) groups. Symptom severity and developmental level were assessed using the Childhood Autism Rating Scale (CARS) and the Gesell Developmental Schedule (GDS). Structural equation modeling (SEM) was used to analyze mediating effects, and machine learning algorithms were employed to assess the predictive importance of developmental dimensions.

**Results:**

In the total sample, SEM revealed a masking effect: the indirect effect of serum 25(OH)D levels on CARS scores via developmental level was negative, whereas the direct effect was positive, offsetting each other and resulting in a non-significant total effect. The mediating effect was attenuated in the VDI group. Machine learning analysis further revealed that language dominates in the NVD group, whereas in the VDI group, the dominance of language is diminished, the predictive importance of personal-social also declines, and fine motor and gross motor show enhanced predictive importance.

**Conclusion:**

Vitamin D insufficiency may weaken the predictive importance of language and personal-social while enhancing motor domains. Sufficient vitamin D was associated with better developmental levels and lower symptom severity, highlighting the potential research relevance of nutritional considerations in studies of ASD symptom severity.

## 1 Introduction

Autism spectrum disorder (ASD) is a neurodevelopmental disorder that emerges in early childhood, characterized by impaired social interaction of varying degrees, restricted interests and stereotyped behaviors. It severely disrupts the social function and quality of life of affected children, and the symptom severity of children with ASD varies significantly (1, 2). Symptom severity, as a core indicator for the clinical management of ASD, is closely associated with children’s long-term prognosis and has been identified as one of the best predictors of future independent living and employment in adulthood (3). Symptom severity was significantly negatively correlated with adaptive behavior, gross motor, fine motor, language and personal-social of developmental level (4), suggesting a close association between ASD symptom severity and developmental level.

The etiology of ASD is multifactorial, involving the complex interactions of genetic, environmental and neurobiological factors (5). Among them, vitamin D status has become a research hotspot due to its potential effects on neurodevelopment (6). Its active form, 1,25(OH)_2_-vitamin D, exerts its functions by binding to the vitamin D receptor (VDR), which is widely distributed in the brain, indicating that vitamin D is involved in important neurobiological processes (7, 8). Studies have reported that vitamin D deficiency may be associated with a variety of neurodevelopmental disorders including ASD (9, 10), and animal studies have demonstrated that vitamin D deficiency could lead to neurodevelopmental abnormalities such as reduced social behavior and impaired learning and memory ability in offspring (11). These findings suggest a certain association between vitamin D status and developmental level, while population studies have not shown a significant correlation between them (12).

A multicenter study in China showed a significant negative correlation between serum 25(OH)D levels and ASD symptom severity (12), whereas several other studies found no significant association (13–15). These findings indicate that the relationship between the two remains complex and inconsistent. The association between serum 25(OH)D levels and ASD symptom severity may not be a simple direct linear relationship; instead, 25(OH)D may indirectly affect symptom severity by influencing children’s neurodevelopment. Whether developmental level, as an important indicator reflecting neurodevelopment in children (16), plays a mediating role between serum 25(OH)D levels and ASD symptom severity remains to be further investigated.

Existing studies on the relationship between serum 25(OH)D levels and ASD have predominantly adopted case-control designs, with limited evidence from diagnosed children, and no study has examined whether vitamin D status modifies the pathway through which 25(OH)D acts on development and symptom severity. To address these gaps, this study employed structural equation modeling (SEM) to analyze the action pathways among serum 25(OH)D levels, developmental level, and symptom severity in children with ASD, and conducted stratified comparisons between vitamin D insufficiency and normal vitamin D groups. Furthermore, given that machine learning approaches have been shown to effectively rank predictors of clinical outcomes based on their relative importance (17), the present study further employed machine learning algorithms for feature importance assessment, so as to evaluate the predictive contribution of each developmental dimension to symptom severity and identify core predictive features under different vitamin D statuses.

## 2 Methods

### 2.1 Participants

A total of 699 children aged 24 ~ 71 months, who were initially diagnosed with ASD at the Department of Children’s Healthcare of a tertiary hospital in Guangxi, China, between January 2022 and December 2023, were selected as study subjects. Inclusion criteria: The children met the diagnostic criteria for ASD as outlined in the Diagnostic and Statistical Manual of Mental Disorders, Fifth Edition (DSM-5) and were diagnosed by senior doctors with many years of experience in developmental-behavioral pediatrics and rich clinical experience. Additionally, they had not received professional and systematic rehabilitation training. Exclusion criteria: (1) Children with Rett syndrome, trisomy 21, fragile X syndrome, genetic metabolic diseases, epilepsy, Attention Deficit Hyperactivity Disorder (ADHD) organic neuropsychiatric disorders, other disintegrative mental disorders, congenital deafness, visual impairment, etc., were excluded. (2) Regular vitamin D supplementation or the use of drugs that affect serum 25(OH)D levels (e.g., antiepileptic drugs and immunosuppressive drugs) in the past 6 months (12). The children’s guardians were informed, agreed to participate in this study, and signed the informed consent form. According to serum 25(OH)D levels, children were categorized into two groups: normal vitamin D (NVD) (> 75 nmol/L) and vitamin D insufficiency (VDI) (< 75 nmol/L) (18). The study was approved by the relevant ethics committee (approval No. 2024-213, approved on 23 October 2024).

#### 2.1.1 Assessment tools

Physical measurements were conducted by trained and qualified specialist nurses from the Department of Children’s Healthcare in our hospital. The same model of infant horizontal measuring bed (<3 years old) or wall-mounted human body meter (≥3 years old) was used for measurement. Body weight and length/height were recorded in kilograms (kg) and centimeters (cm), respectively, both rounded to one decimal place. Before the measurement, children removed their shoes, hats, and diapers and put on lightweight clothing.

ASD symptom assessment: ASD symptom severity was assessed with the Childhood Autism Rating Scale (legacy CARS). This scale comprises 15 items, each rated on a 1 to 4 scale, with a total score of 60 points. A total score below 30 indicates non-ASD; a score of 30 to 36 with fewer than 5 items with a score below 3 indicates mild-to-moderate ASD; a score of 37 to 60 with at least 5 items with a score above 3 indicates severe ASD (19, 20). The Cronbach’s alpha coefficient for this scale was 0.9, demonstrating good reliability (21).

Developmental level assessment: Intellectual and behavioral development was assessed using the Gesell Developmental Schedule (GDS) (22). The GDS covers five sectors, including adaptive behavior, gross motor, fine motor, language, and personal-social. The evaluation results are presented as a developmental quotient (DQ), which was calculated using the formula: DQ = (Developmental Age/Chronological Age) × 100. DQ ≥86 indicates normal. 76–85 indicates borderline, 55–75 indicates mild developmental delay, 40–54 indicates moderate developmental delay, 25–39 indicates severe developmental delay, and <25 indicates extremely severe developmental delay (23).

#### 2.1.2 Quality control

Physical measurements were conducted using standard methods, with instruments calibrated before each measurement and quality supervision provided by on-site inspectors. All professional evaluators possessed evaluation qualifications. The CARS scale assessment was conducted by professionals based on observations of children’s behavior and interviews with parents. The GDS scale assessment was conducted strictly in accordance with operational procedures, using a unified set of administration instructions. Data were entered and checked by two people, and consistency checks were performed.

### 2.2 Determination of serum 25(OH)D levels

Serum 25(OH)D levels were measured by chemiluminescence immunoassay using an automated analyzer (Abbott, USA) with commercially available kits. All procedures were performed strictly following the manufacturer’s instructions.

### 2.3 Statistical methods

Statistical analysis was performed using SPSS (version 26.0; IBM Corp., Armonk, NY, USA). The Shapiro–Wilk test was used to assess normality. For normally distributed quantitative data, results were expressed as mean ± SD, and group comparisons were performed using the independent samples t-test (or Satterthwaite’s t-test for heterogeneous variances). For non-normally distributed data, results were expressed as median (P25, P75), and the Mann–Whitney U test was used for group comparisons. Categorical variables were compared via the chi-square test. For correlation analysis, Pearson’s correlation would have been used for normally distributed data; however, given that most continuous variables in this study were not normally distributed, Spearman’s rank correlation coefficient was applied instead. Differences were deemed statistically significant when *p* < 0.050.

### 2.4 Construction and validation of SEM

To test the mediating hypothesis that serum 25(OH)D levels indirectly affect the severity of ASD symptoms through developmental level, this study constructed an SEM in R (version 4.3.1; R Foundation for Statistical Computing, Vienna, Austria) using the lavaan package for analysis.

#### 2.4.1 Model setting

To control for potential confounding effects, age, height, and weight were included as control variables. Using serum 25(OH)D level as the independent variable and ASD symptom severity (CARS scores) as the dependent variable, the mediating latent variable was developmental level. Developmental level was indicated by the five sectors of GDS: adaptive behavior, gross motor, fine motor, language, and personal-social. In addition, to assess potential multicollinearity among model variables, collinearity diagnostics were performed for all predictors entered into the SEM, with variance inflation factor (VIF) and tolerance calculated. The criteria of VIF < 5 and tolerance > 0.2 were adopted to identify the absence of multicollinearity.

#### 2.4.2 Model fitting and evaluation

The maximum likelihood estimation method was employed for parameter estimation. Standard errors and confidence intervals for the parameter estimates were calculated using the Bootstrap method with 5,000 resamples. Results were considered statistically significant if their 95% confidence intervals excluded 0 and the associated *p*-value was less than 0.05. The evaluation of the measurement model generally adhered to the following criteria: a standardized factor loading of greater than 0.70 was considered ideal, while a value of greater than 0.50 was deemed acceptable. A range of 0.30 to 0.50 was regarded as marginally acceptable (24). The model’s goodness-of-fit was assessed using the following indicators: Comparative Fit Index (CFI), Tucker-Lewis Index (TLI), Root Mean Square Error of Approximation (RMSEA), and Standardized Root Mean Square Residual (SRMR). An acceptable model fit was defined as CFI and TLI values greater than 0.90, and RMSEA and SRMR values less than 0.08. A good fit was characterized by CFI and TLI values greater than 0.95 and RMSEA and SRMR values less than 0.06 (25, 26). Given that no single index should be used in isolation to determine model adequacy, all fit indices were considered collectively when evaluating model fit (25). Model-fitting and path analysis were conducted for the total sample, NVD group and VDI group, respectively.

### 2.5 Machine learning-based feature importance assessment of developmental level

To further validate the predictive contribution of each developmental dimension to ASD symptom severity, this study employed three ensemble learning algorithms for feature importance assessment: Random Forest (RF), Extreme Gradient Boosting (XGBoost), and Light Gradient Boosting Machine (LightGBM).

#### 2.5.1 Algorithm selection and rationale

The three algorithms offer complementary methodological strengths. RF, based on Bagging ensemble and random feature subspace strategy, demonstrates robust resistance to overfitting and directly outputs feature importance derived from impurity reduction. XGBoost incorporates second-order gradient statistics and regularization terms, enabling effective capture of complex nonlinear relationships between developmental level indicators and CARS scores. LightGBM adopts a histogram-based decision tree algorithm and gradient-based one-side sampling technique, offering high training efficiency suitable for moderate sample sizes.

#### 2.5.2 Model configuration and training

The CARS scores served as the target variable, with the five domains of the GDS (adaptive behavior, gross motor, fine motor, language, and personal-social) as predictive features. Missing values were imputed with the mean of the respective variable. The dataset was randomly split into training and test sets at an 8:2 ratio, with the random seed fixed at 42 to ensure reproducibility. Model hyperparameters were set as follows: number of trees = 400 for RF; number of trees = 400, learning rate = 0.05, maximum depth = 4, subsample ratio = 0.8, column sample ratio = 0.8 for XGBoost; number of trees = 400, learning rate = 0.05, number of leaves = 31 for LightGBM. Five-fold cross-validation was applied to assess the stability of the model training process.

#### 2.5.3 Feature importance ranking and stratified analysis

The built-in feature importance scores of each model were extracted. All feature importance values were normalized across models for each feature and aggregated by ranking to obtain the consistent core predictive features. To evaluate the dynamic changes in the predictive weights of developmental level dimensions under different vitamin D statuses, the analysis was further stratified by the normal vitamin D (NVD) group and vitamin D insufficiency (VDI) group. Model training and evaluation were performed using Python (version 3.13.5) with Scikit-learn (version 1.6.1), XGBoost (version 3.2.0), and LightGBM (version 4.6.0).

## 3 Results

### 3.1 Sample characteristics

The VDI group had significantly lower adaptive behavior, gross motor, and personal-social of GDS than the NVD group (P < 0.05), whereas no significant differences were observed in fine motor and language of GDS (P > 0.05). Meanwhile, no significant differences were observed in the distribution of mild-to-moderate and severe ASD cases between the VDI and NVD groups (P > 0.05) (**Table 1**).

**Table 1:** Sample characteristics.

Group	N	GDS					CARS scores	Symptom severity of ASD	
		Adaptive behavior	Gross motor	Fine motor	Language	Personal-social		Mild-to-moderate ASD	Severe ASD
serum 25 (OH)D level									
VDI	434	57 (47,66)	77 (66,85)	65 (55,76)	35 (27,44)	55 (47,64)	38 (34,41)	178 (64.7)	256 (60.4)
NVD	265	61 (51,70)	79 (73,87)	68 (58,77)	36 (30,46)	57 (50,65)	39 (35,41)	97 (35.3)	168 (39.6)
Z score **/***χ*^2^		-3.131	-2.982	-1.523	-1.885	-2.220	-1.319	1.339	
*P value*		0.002	0.003	0.128	0.059	0.026	0.187	0.247	
gender									
male	594	59 (50,67)	78 (69,85)	66 (57,76)	36 (28,45)	56 (48,64)	38 (34,41)		
female	105	58 (47,69)	78 (68,86)	67 (55,74)	36 (28,43)	55 (48,64)	38 (35,42)		
*Z score*		-0.157	-0.125	-0.182	-0.211	-0.519	-0.446		
*P value*		0.875	0.900	0.855	0.833	0.604	0.655		

Data are presented as median (P25, P75). VDI, vitamin D insufficiency; NVD, normal vitamin D; GDS, Gesell Developmental Schedules; CARS, Childhood Autism Rating Scale; Group comparisons were performed using the Mann–Whitney U test, and for categorical variables using the chi-square test.

### 3.2 Spearman’s correlation analysis

Serum 25(OH)D levels were significantly negatively correlated with age, height, and weight (P < 0.001) and significantly positively correlated with adaptive behavior, gross motor, fine motor, language, and personal-social of GDS (P < 0.05). CARS scores were significantly negatively correlated with age, height, weight, adaptive behavior, gross motor, fine motor, language, and personal-social of GDS (P < 0.01) (**Table 2**).

**Table 2:** Spearman’s correlation analysis of serum 25(OH)D levels with clinical characteristics in children with ASD.

Variable	Serum 25(OH)D level	Age	Height	Weight	BMI	Adaptive behavior	Gross motor	Fine motor	Language	Personal-social	CARS scores
serum 25(OH)D level		-0.138	-0.192	-0.172	-0.016	0.169	0.181	0.107	0.080	0.109	0.040
*P* value		<0.001	<0.001	<0.001	0.679	<0.001	<0.001	0.005	0.034	0.004	0.288
*CARS scores*	0.040	-0.133	-0.152	-0.122	0.021	-0.345	-0.097	-0.255	-0.462	-0.303	
*P* value	0.288	<0.001	<0.001	0.001	0.573	<0.001	<0.011	<0.001	<0.001	<0.001	

Values shown are Spearman's correlation coefficients. CARS, Childhood Autism Rating Scale.

### 3.3 SEM

Model-fitting indices showed that for the total sample: CFI = 0.975, TLI = 0.937, RMSEA = 0.077, SRMR = 0.044; for the NVD group: CFI = 0.958, TLI = 0.905, RMSEA = 0.089, SRMR = 0.058; for the VDI group: CFI = 0.981, TLI = 0.952, RMSEA = 0.069, SRMR = 0.040 (**Table 3**). In the NVD group, the RMSEA value of 0.089 was at a marginal level relative to the conventional threshold of 0.08, but remained within broadly acceptable limits, and the other fit indices for this group were within acceptable ranges (CFI = 0.958, TLI = 0.905, SRMR = 0.058). Collectively, all model-fitting indices fell within acceptable ranges.

**Table 3:** Goodness-of-fit indices of the structural equation models for the total sample and vitamin D subgroups.

Fit indices	Allowable range	Total sample	NVD group	VDI group
GFI	>0.90	0.975	0.958	0.981
TLI	>0.90	0.937	0.905	0.952
*RMSEA*	<0.08	0.077	0.089	0.069
SRMR	<0.08	0.044	0.058	0.040

NVD, normal vitamin D group; VDI, vitamin D insufficiency group; SRMR, standardized root mean square residual; RMSEA, root mean square error of approximation; CFI, comparative fit index; TLI, Tucker-Lewis index.

#### 3.3.1 Measurement model

**Figure 1:**
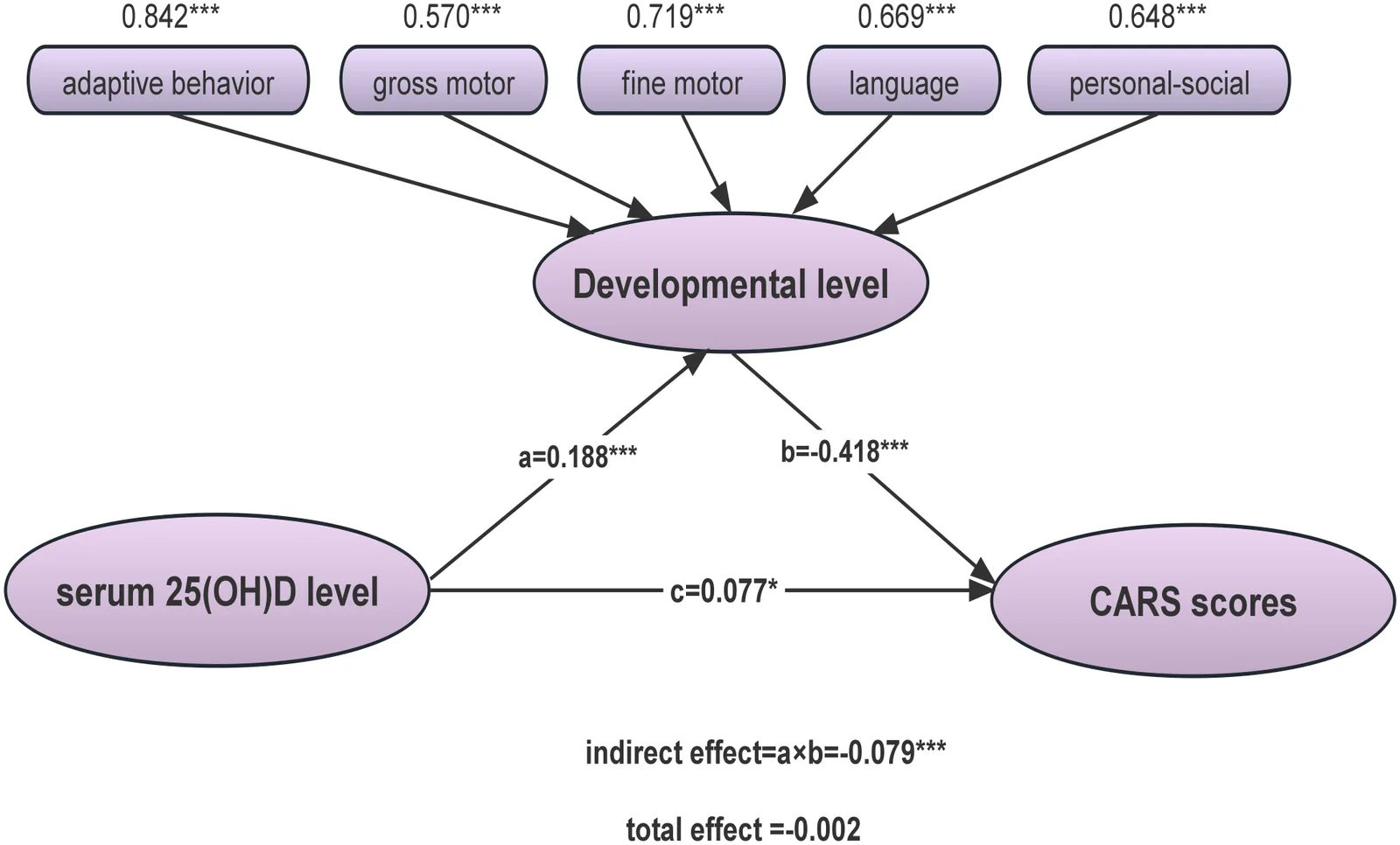
Path diagram for the total sample with standardized coefficients. All paths are significant at *P < 0.05, **P < 0.01, ***P < 0.001. Age, height, and weight were included as control variables.

**Figure 2:**
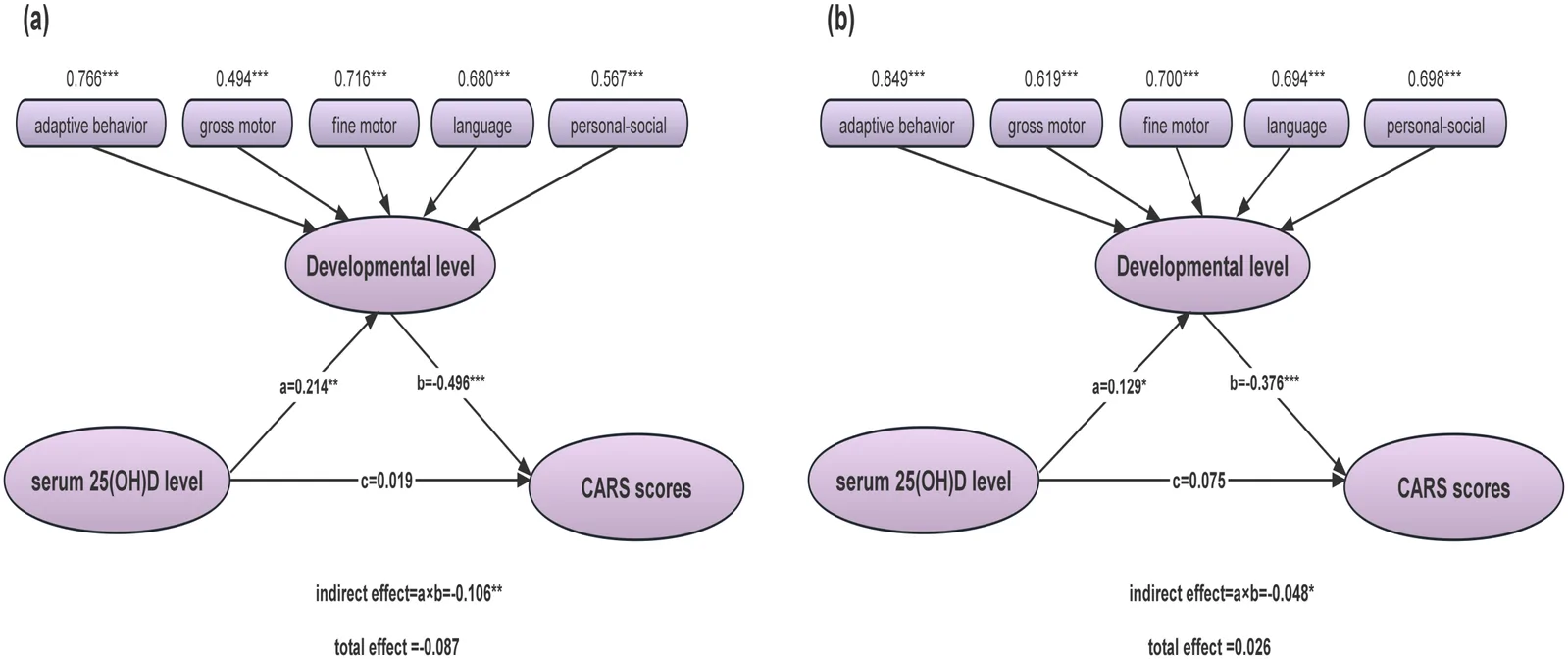
Path diagrams for vitamin D subgroups with standardized coefficients. **(a)** Normal vitamin D (NVD) group; **(b)** Vitamin D insufficiency (VDI) group. All paths shown are significant at *P < 0.05, **P < 0.01, ***P < 0.001. Age, height, and weight were included as control variables in both models.

Collinearity diagnostics revealed that serum 25(OH)D levels (VIF = 1.080) and the five developmental domains all had VIF values below 2.7, indicating no multicollinearity among core variables. The standardized loadings of all observed variables on their respective latent variables were significant (P < 0.001). In the total sample, the loadings for each developmental level dimension ranged from 0.570 to 0.842 (**Table 4**, **Figure 1**). In the NVD group, the loadings for developmental level ranged from 0.494 to 0.766 (**Table 5**, **Figure 2a**). In the VDI group, the loadings for developmental level ranged from 0.619 to 0.849 (**Table 6**, **Figure 2b**).

**Table 4:** Path coefficients of the structural equation model for the total sample.

Variables	B	95% Confidence Interval		Z	P	β
		Lower	Upper			
measurement model						
adaptive behavior→developmental level	1.000	1.000	1.000			0.842
gross motor→developmental level	0.613	0.526	0.706	13.400	<0.001	0.570
fine motor→developmental level	0.964	0.868	1.066	19.195	<0.001	0.719
language→developmental level	0.779	0.672	0.921	12.413	<0.001	0.669
personal-social→developmental level	0.691	0.601	0.791	14.174	<0.001	0.648
structural model						
serum 25(OH)D level→developmental level	0.108	0.061	0.155	4.520	<0.001	0.188
developmental level→CARS scores	-0.257	-0.318	-0.203	-8.800	<0.001	-0.418
serum 25(OH)D level→CARS scores	0.027	0.006	0.049	2.441	0.015	0.077
mediating effect						
indirect effect	-0.028	-0.042	-0.015	-4.082	<0.001	-0.079
direct effect	0.027	0.006	0.049	2.441	0.015	0.077
total effect	-0.001	-0.024	0.024	-0.045	0.964	-0.002

*Notes*: B, unstandardized regression coefficient; β, standardized regression coefficient; 95% CI, 95% confidence interval for the unstandardized coefficient (B); z: z-value; *p*: *p*-value. Indirect effect = serum 25(OH)D level → developmental level → CARS scores; direct effect = serum 25(OH)D level → CARS scores; total effect = indirect effect + direct effect. Bootstrap with 5000 resamples was used to calculate 95% confidence intervals. Age, height, and weight were included as control variables.Effect size classification for mediation paths followed established simulation benchmarks (27): standardized single path coefficient β = 0.14 = small effect, β = 0.26 = small-to-medium effect, β = 0.39 = medium effect, β = 0.59 = large effect. For indirect mediated effect (product of two standardized paths): absolute value <0.05 = trivial minimal effect, 0.05–0.10 = small effect, 0.10–0.20 = small-to-medium effect.

**Table 5:** Path coefficients of the structural equation model for the normal vitamin D (NVD) group.

Variables	B	95% Confidence Interval		Z	P	β
		Lower	Upper			
measurement model						
adaptive behavior→developmental level	1.000	1.000	1.000			0.766
gross motor→developmental level	0.578	0.414	0.750	6.727	<0.001	0.494
fine motor→developmental level	1.061	0.857	1.298	9.391	<0.001	0.716
language→developmental level	0.986	0.684	1.598	3.992	<0.001	0.680
personal-social→developmental level	0.674	0.471	0.976	5.105	<0.001	0.567
structural model						
serum 25(OH)D level→developmental level	0.160	0.053	0.275	2.793	0.005	0.214
developmental level→CARS scores	-0.334	-0.504	-0.213	-4.488	<0.001	-0.496
serum 25(OH)D level →CARS scores	0.010	-0.037	0.052	0.422	0.673	0.019
mediating effect						
indirect effect	-0.053	-0.098	-0.018	-2.599	0.009	-0.106
direct effect	0.010	-0.037	0.052	0.422	0.673	0.019
total effect	-0.044	-0.101	0.006	-1.599	0.110	-0.087

*Notes*: B, unstandardized regression coefficient; β, standardized regression coefficient; 95% CI, 95% confidence interval for the unstandardized coefficient (B); z: z-value; *p*: *p*-value. Indirect effect = serum 25(OH)D level → developmental level → CARS scores; direct effect = serum 25(OH)D level → CARS scores; total effect =indirect effect + direct effect. Bootstrap with 5000 resamples was used to calculate 95% confidence intervals. Age, height, and weight were included as control variables. Effect size classification for mediation paths followed established simulation benchmarks (27): standardized single path coefficient β = 0.14 = small effect, β = 0.26 = small-to-medium effect, β = 0.39 = medium effect, β = 0.59 = large effect. For indirect mediated effect (product of two standardized paths): absolute value <0.05 = trivial minimal effect, 0.05–0.10 = small effect, 0.10–0.20 = small-to-medium effect.

**Table 6:** Path coefficients of the structural equation model for the vitamin D insufficiency (VDI) group.

Variables	B	95% Confidence Interval		Z	P	β
		Lower	Upper			
measurement model						
adaptive behavior→developmental level	1.000	1.000	1.000			0.849
gross motor→developmental level	0.662	0.552	0.785	11.145	<0.001	0.619
fine motor→developmental level	0.934	0.831	1.042	17.301	<0.001	0.700
language→developmental level	0.743	0.631	0.874	11.952	<0.001	0.694
personal-social→developmental level	0.735	0.585	0.895	9.344	<0.001	0.698
structural model						
serum 25(OH)D level→developmental level	0.130	0.024	0.240	2.370	0.018	0.129
developmental level→CARS scores	-0.231	-0.296	-0.168	-6.993	<0.001	-0.376
serum 25(OH)D level →CARS scores	0.046	-0.007	0.105	1.616	0.106	0.075
mediating effect						
indirect effect	-0.030	-0.058	-0.005	-2.261	0.024	-0.048
direct effect	0.046	-0.007	0.105	1.616	0.106	0.075
total effect	0.016	-0.038	0.074	0.564	0.573	0.026

*Notes*: B, unstandardized regression coefficient; β, standardized regression coefficient; 95% CI, 95% confidence interval for the unstandardized coefficient (B); z: z-value; *p*: *p*-value. Indirect effect = serum 25(OH)D level → developmental level → CARS scores; direct effect = serum 25(OH)D level → CARS scores; total effect = indirect effect + direct effect. Bootstrap with 5000 resamples was used to calculate 95% confidence intervals. Age, height, and weight were included as control variables. Effect size classification for mediation paths followed established simulation benchmarks (27): standardized single path coefficient β = 0.14 = small effect, β = 0.26 = small-to-medium effect, β = 0.39 = medium effect, β = 0.59 = large effect. For indirect mediated effect (product of two standardized paths): absolute value <0.05 = trivial minimal effect, 0.05–0.10 = small effect, 0.10–0.20 = small-to-medium effect.

#### 3.3.2 Structural model

After controlling for age, height, and weight, 25(OH)D exerted a significant positive effect on developmental level (B = 0.108 (95% CI: 0.061–0.155), β = 0.188, small-medium effect, P < 0.001). Developmental level had a significant negative effect on CARS scores (B = −0.257 (95% CI: −0.318–−0.203), β = −0.418, medium effect, P < 0.001). Mediation analysis results showed that the indirect effect of developmental level between 25(OH)D and CARS scores was significant (B = −0.028, 95% CI = −0.042–−0.015, β = −0.079, small indirect effect, P < 0.001). The direct effect of 25(OH)D on CARS scores was significant (B = 0.027, 95% CI = 0.006–0.049, β = 0.077, small effect, P < 0.05), which was opposite in direction to the indirect effect. The two effects offset each other, resulting in a non-significant total effect (B = −0.001 (95% CI: −0.024–0.024), β = −0.002, trivial effect, P > 0.05) (**Table 4**, **Figure 1**).

#### 3.3.3 Subgroup analysis

In the NVD group, serum 25(OH)D levels had a significant positive effect on developmental level (B = 0.160 (95% CI: 0.053–0.275), β = 0.214, small-medium effect, P < 0.01). Developmental level exerted a significant negative effect on CARS scores (B = −0.334 (95% CI: −0.504–−0.213), β = −0.496, medium effect, P < 0.001). The indirect effect of developmental level between serum 25(OH)D levels and CARS scores was significant (B = −0.053, 95% CI = −0.098–−0.018, β = −0.106, small-medium indirect effect, P < 0.01) (**Table 5**, **Figure 2a**).

In the VDI group, compared with the NVD group, the path relationships changed significantly: the positive effect of serum 25(OH)D levels on developmental level was attenuated (B = 0.130 (95% CI: 0.024–0.240), β = 0.129, small effect, P < 0.05). Meanwhile, the negative effect of developmental level on CARS scores was also attenuated (B = −0.231 (95% CI: −0.296–−0.168), β = −0.376, medium effect, P < 0.001). The indirect effect of developmental level between serum 25(OH)D levels and CARS scores was attenuated but significant (B = −0.030, 95% CI: −0.058–−0.005, β = −0.048, trivial indirect effect, P < 0.05) (**Table 6**, **Figure 2b**).

#### 3.3.4 Subgroup comparison of model explained variance (R^2^)

The coefficient of determination R^2^ was used to quantify the proportion of variance in CARS scores jointly explained by serum 25(OH)D levels and the latent variable developmental level, after adjusting for confounding variables including age, height and weight. The adjusted model coefficient of determination was R^2^ = 0.173 for the total sample, indicating that the model explained 17.3% of the variance in CARS scores. Subgroup analyses revealed that the adjusted R^2^ was 0.264 in the NVD group, accounting for 26.4% of the variance in CARS scores; the adjusted R^2^ was 0.139 in the VDI group, explaining only 13.9% of the variance in CARS scores. There was a 12.5 percentage point difference in model explained variance between the two groups (**Table 7**). The above intergroup difference in explained variance is consistent with the findings from stratified SEM, tentatively suggesting that vitamin D insufficiency may attenuate the explanatory power of developmental level regarding ASD symptom severity.

**Table 7:** Adjusted R² of regression models controlling for age, height and weight.

Group	Adjusted R²	Explained variance of CARS scores|
Total sample	0.173	17.3%
NVD group	0.264	26.4%
VDI group	0.139	13.9%

All models were adjusted for age, height and weight. Adjusted R² represents the proportion of variance in CARS scores jointly explained by serum 25(OH)D and latent developmental level. NVD, normal vitamin D group; VDI, vitamin D insufficiency group.

### 3.4 Comparison of predictive importance of developmental dimensions stratified by vitamin D status

**Figure 3:**
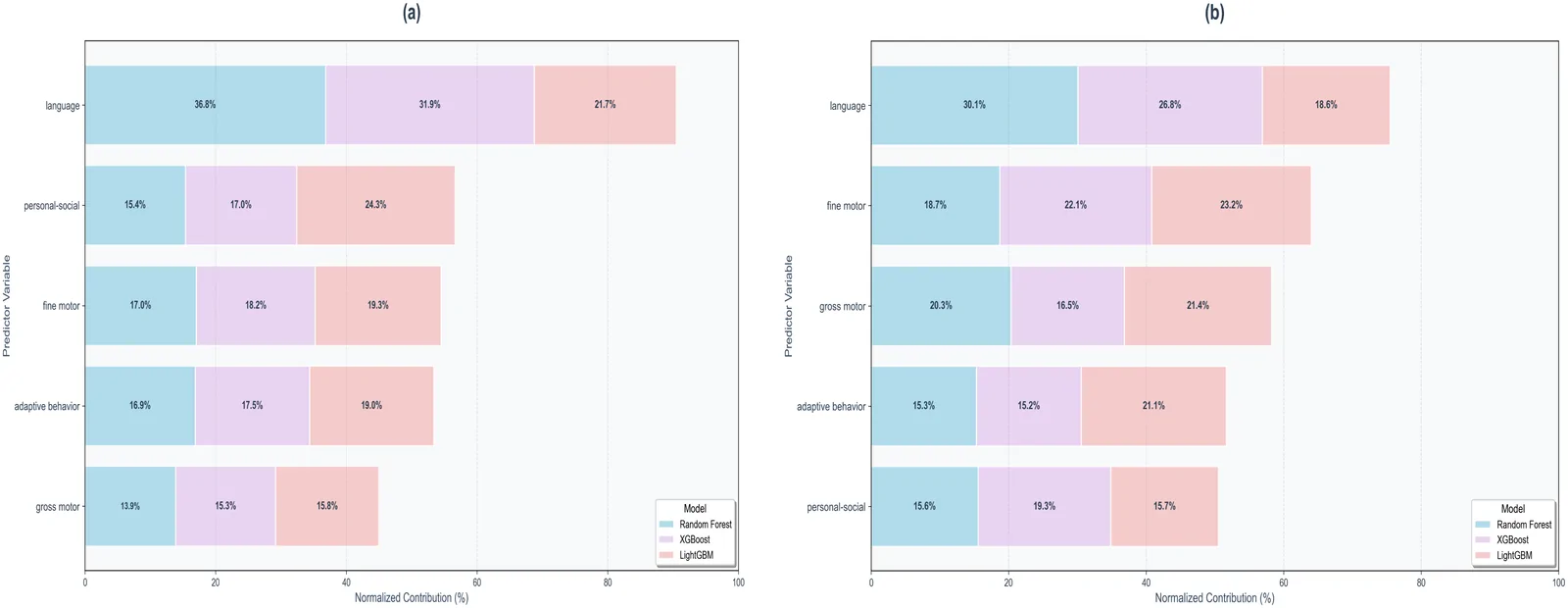
Comparison of aggregated feature importance rankings from three ensemble machine learning models for predicting ASD symptom severity. **(a)** Normal vitamin D (NVD) group. **(b)** Vitamin D insufficiency (VDI) group.

In the NVD group, based on the aggregated ranking across the three models, the order of predictive importance for each domain was as follows: language (90.4%) > personal-social (56.7%) > fine motor (54.5%) > adaptive behavior (53.4%) > gross motor (45.0%). The language domain was approximately 1.6 times that of the second-ranked feature, occupying a core predictive position (**Figure 3a**).

In the VDI group, the predictive structure was changed: language decreased to 75.5%, with diminished dominance; fine motor increased to 64.0%, rising to the second position; gross motor increased to 58.2%, rising to the third position; adaptive behavior (51.6%) remained in the fourth position; personal-social decreased to 50.6%, falling to the fifth position (**Figure 3b**).

## 4 Discussion

ASD is a neurodevelopmental disorder with highly heterogeneous clinical manifestations, and its symptom severity is closely associated with the developmental level of children. This study explored the complex mechanisms underlying the effects of serum 25(OH)D levels on the developmental level and symptom severity based on a cohort of children with confirmed ASD.

This study observed that children with ASD in the VDI group had significantly lower adaptive behavior, gross motor, and personal-social of GDS than those in the NVD group, with no significant differences found in fine motor and language of GDS. This indicates that the impact of vitamin D insufficiency on the neurodevelopment of children with ASD is not global. A study reported that the abnormal detection rates of aggressive, impulsive, tantrum and oppositional defiant behaviors in the overt behavioral domain of emotional and social development, as well as severe mood swings, poor soothability and inattention in the dysregulation domain, were significantly higher in ASD children with vitamin D insufficiency (28), which confirmed the significant decline in personal-social, adaptive behavior and gross motor of GDS in the VDI group in this study. Animal studies demonstrated that vitamin D insufficiency could lead to reduced social behavior and impaired learning and memory abilities in offspring, accompanied by the downregulated expression of the cerebellar Drd2 gene that contributes to the improvement of gross motor of GDS (11). These findings further validated the results of our present study that ASD children in the VDI group had significantly lower adaptive behavior, gross motor, and personal-social of GDS than those in the NVD group.

Inconsistent conclusions have been reached regarding the association between serum 25(OH)D levels and ASD symptom severity. Through SEM, the present study revealed complex pathways underlying this association. After controlling for age, height, and weight, results of the SEM for the total sample showed that serum 25(OH)D levels exerted a significant positive direct effect on ASD symptom severity, small effect; meanwhile, serum 25(OH)D levels exerted a significant negative indirect effect on ASD symptom severity through developmental level, small indirect effect. The indirect effect was opposite in direction and similar in magnitude to the direct effect, and the two effects offset each other, leading to a non-significant total effect with trivial effect. This resulted in a statistical masking effect (29). This finding explains why no significant association was observed between serum 25(OH)D levels and ASD symptom severity in the Spearman correlation analysis, and the positive direct effect only became evident when developmental level was incorporated into the model.

Subgroup analysis of the SEM revealed that in the NVD group, serum 25(OH)D levels had a significant positive effect on developmental level, small-medium effect, which in turn exerted a significant negative effect on ASD symptom severity, medium effect, forming a protective mediating pathway suggesting that higher serum 25(OH)D levels may be associated with better developmental level, which in turn may contribute to milder symptoms. In the VDI group, the positive effect of serum 25(OH)D levels on developmental level was attenuated to 60.3% of that in the NVD group, small effect, the negative effect of developmental level on ASD symptom severity was attenuated to 75.8% of that in the NVD group, medium effect, and the corresponding mediating effect was only 45.3% of that in the NVD group, trivial indirect effect. These results indicate that the correlational link between serum 25(OH)D levels and developmental level, as well as the correlational link between developmental level and ASD symptom severity, tends to be weaker under conditions of vitamin D insufficiency, thereby contributing to the attenuation of the mediating effect. Consistent with the attenuated path coefficients, the adjusted R² values further verified that the combined predictive power of 25(OH)D and developmental level for CARS scores was markedly lower in the VDI group (13.9%) relative to the NVD group (26.4%), with a 12.5% gap in explained variance between subgroups.

The results of the subgroup analysis in the present study are directly validated by clinical experimental evidence. A study by Jia et al. (30) found that the core symptoms of three children with ASD improved significantly when their serum 25(OH)D levels increased above 40.0 ng/mL after supplementation, which was consistent with the positive effect of serum 25(OH)D levels on developmental level in the present model; in contrast, their core symptoms deteriorated again and developed more severe symptoms when supplementation was discontinued and serum 25(OH)D levels decreased below 30.0 ng/mL, which further supports the finding that lower serum 25(OH)D levels correlate with lower developmental level and more severe symptoms.

From a neurobiological perspective, the attenuation of the mediating effect caused by vitamin D insufficiency may be achieved through multiple pathways jointly. The vitamin D-MMP-9-perineuronal net regulatory axis proposed by Mayne et al. (31) indicated that vitamin D insufficiency can upregulate the expression of matrix metalloproteinase-9 (MMP-9), destroy the structural integrity of perineuronal nets (PNNs), thereby impairing the maturation and function of parvalbumin-positive interneurons and leading to a decrease in neural network plasticity. Such plasticity impairment may further affect children’s developmental level and exacerbate ASD symptoms. At the synaptic function level, Kasatkina et al. (32) demonstrated that vitamin D insufficiency impairs neuronal plasticity and information integration ability by inducing oxidative stress, synaptic vesicle fusion disorders and excitation/inhibition imbalance, thus attenuating the positive effect of serum 25(OH)D levels on developmental level. Meanwhile, vitamin D insufficiency enhances the sensitivity of mGluR7 receptors and interferes with GABAergic inhibitory transmission, resulting in a reduction in the functional reserve of the nervous system. This makes ASD symptoms more likely to manifest at the same developmental level, that is, the negative effect of developmental level on symptom severity is attenuated. The synergistic effect of these two pathways may underpin the attenuated protective mediating effect observed in the vitamin D insufficiency group in the present study. These mechanistic frameworks provide potential biological plausibility for the cross-sectional associations identified herein.

The machine learning feature importance analysis further elucidated the differential predictive contributions of specific developmental domains to ASD symptom severity under varying vitamin D statuses. In the NVD group, language emerged as the dominant predictor, with an aggregated importance score substantially exceeding that of all other domains, consistent with prior evidence that early language ability is a strong predictor of long-term clinical outcomes in children with ASD (33). In the VDI group, the predictive structure was changed: the dominance of language was diminished, and personal-social also declined in predictive importance, whereas fine motor and gross motor gained greater predictive weight. The diminished predictive weight of language and personal-social domains may be attributable to the impaired neural network plasticity and excitation/inhibition imbalance caused by vitamin D insufficiency, which have been shown to disproportionately affect higher-order cortical functions such as language and social cognition (31, 32). Conversely, the increased importance of motor domains may reflect the differential vulnerability of motor-related neural pathways to vitamin D insufficiency, as suggested by prior evidence linking vitamin D insufficiency to motor impairments in children with ASD (11, 28). Taken together, these findings suggest that vitamin D insufficiency not only attenuates the mediating pathway from serum 25(OH)D levels to symptom severity via developmental level, as demonstrated in the SEM, but also reshapes the relative importance of specific developmental dimensions in predicting core symptoms.

### 4.1 Limitations

The sample was sourced from a single hospital, potentially leading to selection bias and limiting the generalizability of the results. Furthermore, potential confounding factors such as family socioeconomic status, genetic background, sunlight exposure, and season of blood collection were not controlled for, which may have exerted a certain impact on the results. Children with global intellectual disability (ID) were not excluded at enrollment. Notably, cross-sectional data cannot rule out reverse causation: severe ASD symptoms may reduce outdoor sunlight exposure and lower 25(OH)D levels, as pointed out in a recent critical systematic review (34). Accordingly, the associations observed in this study cannot be interpreted as unidirectional causal effects and should be explained with great caution. In addition, the machine learning analysis lacked external validation, and future multicenter, prospective studies with larger cohorts are warranted. Subgroup SEM results are only exploratory conclusions.

## 5 Conclusion

In the total sample, using SEM, this study identifies a masking effect of serum 25(OH)D levels on ASD symptom severity. Specifically, serum 25(OH)D levels exert a negative indirect effect on symptom severity via developmental level, yet a positive direct effect on symptom severity; the offsetting of these two effects results in a non-significant total effect. The mediating effect was attenuated in the VDI group, thereby explaining the inconsistencies in previous research conclusions. Machine learning analysis further revealed that language dominates in the NVD group, whereas in the VDI group, the dominance of language is diminished, the predictive importance of personal-social also declines, and fine motor and gross motor show enhanced predictive importance, suggesting that vitamin D insufficiency may weaken the predictive importance of language and personal-social while enhancing that of motor domains, and that maintaining sufficient vitamin D may be associated with better developmental levels and lower symptom severity, highlighting the potential research relevance of nutritional considerations in studies of ASD symptom severity.

## Data Availability

The original contributions presented in the study are included in the article/supplementary material. Further inquiries can be directed to the corresponding authors.
