# Supplementary material for: A masking effect of vitamin D on ASD symptom severity: mediation by developmental level and altered predictive features in vitamin D insufficiency
Source: Front Psychiatry. 2026 Aug 28;17:1900932. doi: 10.3389/fpsyt.2026.1900932 (PMC13563125; doi:10.3389/fpsyt.2026.1900932)
Supplement: Supplementary file 1 [file DataSheet1.pdf]

Structural equation model codes for the total sample

```
# Load required R packages
library(tidyverse)          # Data manipulation and visualization
library(lavaan)             # Structural equation modeling
library(semPlot)            # Plotting SEM paths
library(readxl)             # Reading Excel files

# Read data file (please ensure the file path is correct)
data <- read_excel('C:/age at first diagnosis/vitamin/25(OH)D/Research data/total sample.xlsx')

# Check data - confirm variables exist and have correct format
str(data)          # View data structure
head(data)         # View first few rows
# Construct structural equation model with age, D, height, weight as covariates
model <- '
    # Measurement model - fix first loading to 1
    Developmental =~ 1*Adaptive + Gross_Motor + Fine_Motor + Language + Social_Skills

    # Structural model: Developmental and CARS regressed on all covariates
    Developmental ~ a1*Age + vd1*D + h1*Height + w1*Weight
    CARS ~ b1*Developmental + cp*Age + cvd*D + h2*Height + w2*Weight

    # Covariances among all exogenous variables (control variables)
    Age ~~ D + Height + Weight
    D ~~ Height + Weight
    Height ~~ Weight

    # Define indirect and total effects for D (your main interest)
    indirect_d_dev := vd1 * b1          # D -> Developmental -> CARS
    direct_d := cvd                     # D -> CARS direct
    total_d := cvd + indirect_d_dev     # total effect of D

    # Indirect and total effects for Age (optional, keep for completeness)
    indirect_age_dev := a1 * b1
    direct_age := cp
    total_age := cp + indirect_age_dev

    # Optional: indirect effects for Height and Weight (if you want to report)
    indirect_height_dev := h1 * b1
    indirect_weight_dev := w1 * b1

    # Residual correlations (based on theory or modification indices)
    Adaptive ~~ Fine_Motor
    Gross_Motor ~~ Fine_Motor
```

```
    Language ~~ Social_Skills
    Gross_Motor ~~ Social_Skills
    Fine_Motor ~~ Social_Skills
    Gross_Motor ~~ Height      # Added based on modification index (MI = 12.010)
    Language ~~ Height         # Added based on modification index (MI = 13.075); theoretically
more plausible

'

# Fit model with RAM representation and bootstrap
fit <- sem(model,
           data = data,
           estimator = "ML",
           missing = "ML",                   # Full information maximum likelihood for
missing
           representation = "RAM",           # RAM representation to handle residual
correlations
           se = "bootstrap",
           bootstrap = 5000)                 # Increase to 500 or 1000 for stable SE

# View summary with fit measures, standardized coefficients, CIs
summary(fit,
        standardized = TRUE,
        fit.measures = TRUE,
        rsquare = TRUE,
        ci = TRUE)

# Check modification indices (optional, for potential improvements)
modindices(fit, sort = TRUE, minimum.value = 10)

# Plot path diagram (standardized coefficients)
semPaths(fit,
         what = "std",
         layout = "tree",
         fade = FALSE,
         residuals = TRUE,
         nCharNodes = 0,
         edge.label.cex = 0.9,
         sizeMan = 8,
         sizeLat = 10,
         edge.color = "black",
         nodeLabels = c("Age", "D", "Height", "Weight",
                        "Adaptive", "Gross_Motor", "Fine_Motor",
                        "Language", "Social_Skills", "CARS",
```

```
                        "Developmental"),
label.cex = 1.1,
curvePivot = TRUE)
```

Structural equation model code for the normal vitamin D (NVD) group

```
# Load required R packages
library(tidyverse)          # Data manipulation and visualization
library(lavaan)             # Structural equation modeling
library(semPlot)            # Plotting SEM paths
library(readxl)             # Reading Excel files

# Read data file (please ensure the file path is correct)
data <- read_excel('C:/age at first diagnosis/vitamin/25(OH)D/Research data/normal vitamin D (NVD).xlsx')

# Check data - confirm variables exist and have correct format
str(data)          # View data structure
head(data)         # View first few rows

# Construct structural equation model with age, D, height, weight as covariates
model <- '
    # Measurement model - fix first loading to 1
    Developmental =~ 1*Adaptive + Gross_Motor + Fine_Motor + Language + Social_Skills

    # Structural model: Developmental and CARS regressed on all covariates
    Developmental ~ a1*Age + vd1*D + h1*Height + w1*Weight
    CARS ~ b1*Developmental + cp*Age + cvd*D + h2*Height + w2*Weight

    # Covariances among all exogenous variables (control variables)
    Age ~~ D + Height + Weight
    D ~~ Height + Weight
    Height ~~ Weight

    # Define indirect and total effects for D (your main interest)
    indirect_d_dev := vd1 * b1          # D -> Developmental -> CARS
    direct_d := cvd                     # D -> CARS direct
    total_d := cvd + indirect_d_dev     # total effect of D

    # Indirect and total effects for Age (optional, keep for completeness)
    indirect_age_dev := a1 * b1
    direct_age := cp
    total_age := cp + indirect_age_dev

    # Optional: indirect effects for Height and Weight (if you want to report)
    indirect_height_dev := h1 * b1
    indirect_weight_dev := w1 * b1

    # Add reasonable residual correlations (based on theory or data exploration)
```

```
    # Correlations within developmental domains
    Adaptive ~~ Fine_Motor
    Language ~~ Social_Skills
    Gross_Motor ~~ Fine_Motor
    Gross_Motor ~~ Social_Skills
    Gross_Motor ~~ Height    # Added based on modification index (MI = 12.853)
'

# Fit model with RAM representation and bootstrap
fit <- sem(model,
           data = data,
           estimator = "ML",
           missing = "ML",                    # Full information maximum likelihood for
missing
           representation = "RAM",           # RAM representation to handle residual
correlations
           se = "bootstrap",
           bootstrap =5000)                   # Increase to 500 or 1000 for stable SE

# View summary with fit measures, standardized coefficients, CIs
summary(fit,
        standardized = TRUE,
        fit.measures = TRUE,
        rsquare = TRUE,
        ci = TRUE)

# Check modification indices (optional, for potential improvements)
modindices(fit, sort = TRUE, minimum.value = 10)

# Plot path diagram (standardized coefficients)
semPaths(fit,
         what = "std",
         layout = "tree",
         fade = FALSE,
         residuals = TRUE,
         nCharNodes = 0,
         edge.label.cex = 0.9,
         sizeMan = 8,
         sizeLat = 10,
         edge.color = "black",
         nodeLabels = c("Age", "D", "Height", "Weight",
                        "Adaptive", "Gross_Motor", "Fine_Motor",
                        "Language", "Social_Skills", "CARS",
                        "Developmental"),
```

```
label.cex = 1.1,
curvePivot = TRUE)
```

Structural equation model code for the vitamin D insufficiency (VDI) group

```
# Load required R packages
library(tidyverse)          # Data manipulation and visualization
library(lavaan)              # Structural equation modeling
library(semPlot)             # Plotting SEM paths
library(readxl)              # Reading Excel files

# Read data file (please ensure the file path is correct)
data <- read_excel('C:/age at first diagnosis/vitamin/25(OH)D/Research data/vitamin D insufficiency (VDI).xlsx')

# Check data - confirm variables exist and have correct format
str(data)           # View data structure
head(data)          # View first few rows
# Construct structural equation model with age, D, height, weight as covariates
model <- '
    # Measurement model - fix first loading to 1
    Developmental =~ 1*Adaptive + Gross_Motor + Fine_Motor + Language + Social_Skills

    # Structural model: Developmental and CARS regressed on all covariates
    Developmental ~ a1*Age + vd1*D + h1*Height + w1*Weight
    CARS ~ b1*Developmental + cp*Age + cvd*D + h2*Height + w2*Weight

    # Covariances among all exogenous variables (control variables)
    Age ~~ D + Height + Weight
    D ~~ Height + Weight
    Height ~~ Weight

    # Define indirect and total effects for D (your main interest)
    indirect_d_dev := vd1 * b1          # D -> Developmental -> CARS
    direct_d := cvd                     # D -> CARS direct
    total_d := cvd + indirect_d_dev     # total effect of D

    # Indirect and total effects for Age (optional, keep for completeness)
    indirect_age_dev := a1 * b1
    direct_age := cp
    total_age := cp + indirect_age_dev

    # Optional: indirect effects for Height and Weight (if you want to report)
    indirect_height_dev := h1 * b1
    indirect_weight_dev := w1 * b1

    # Add reasonable residual correlations (based on theory or data exploration)
    # Correlations within developmental domains
```

```
    Adaptive ~~    Fine_Motor + Social_Skills
    Gross_Motor ~~ Fine_Motor
    Language ~~ Social_Skills
    Gross_Motor ~~ Social_Skills
    Fine_Motor ~~ Social_Skills
    Language ~~ Height    # Added based on modification index (MI = 10.522)

'

# Fit model with RAM representation and bootstrap
fit <- sem(model,
           data = data,
           estimator = "ML",
           missing = "ML",                       # Full information maximum likelihood for
missing
           representation = "RAM",               # RAM representation to handle residual
correlations
           se = "bootstrap",
           bootstrap =5000)                 # Increase to 500 or 1000 for stable SE

# View summary with fit measures, standardized coefficients, CIs
summary(fit,
        standardized = TRUE,
        fit.measures = TRUE,
        rsquare = TRUE,
        ci = TRUE)

# Check modification indices (optional, for potential improvements)
modindices(fit, sort = TRUE, minimum.value = 10)

# Plot path diagram (standardized coefficients)
semPaths(fit,
         what = "std",
         layout = "tree",
         fade = FALSE,
         residuals = TRUE,
         nCharNodes = 0,
         edge.label.cex = 0.9,
         sizeMan = 8,
         sizeLat = 10,
         edge.color = "black",
         nodeLabels = c("Age", "D", "Height", "Weight",
                        "Adaptive", "Gross_Motor", "Fine_Motor",
                        "Language", "Social_Skills", "CARS",
```

```
                     "Developmental"),
label.cex = 1.1,
curvePivot = TRUE)
```
