# Supplementary material for: A masking effect of vitamin D on ASD symptom severity: mediation by developmental level and altered predictive features in vitamin D insufficiency
Source: Front Psychiatry. 2026 Aug 28;17:1900932. doi: 10.3389/fpsyt.2026.1900932 (PMC13563125; doi:10.3389/fpsyt.2026.1900932)
Supplement: Supplementary file 2 [file DataSheet2.pdf]

**Machine learning feature importance ranking code for the total sample**

```
# -*- coding: utf-8 -*-
"""
Created on Sat Apr 18 04:20:37 2026

"""

import argparse
from pathlib import Path
from typing import Iterable

import matplotlib.pyplot as plt
import numpy as np
import pandas as pd
import seaborn as sns
from sklearn.ensemble import RandomForestRegressor
from sklearn.inspection import permutation_importance
from sklearn.metrics import mean_absolute_error, mean_squared_error, r2_score
from sklearn.model_selection import KFold, cross_validate, train_test_split

try:
    from xgboost import XGBRegressor
except Exception:
    XGBRegressor = None

try:
    from lightgbm import LGBMRegressor
except Exception:
    LGBMRegressor = None

try:
    import shap
except Exception:
    shap = None


plt.rcParams["font.sans-serif"] = ["Arial"]
plt.rcParams["axes.unicode_minus"] = False

# Soft color palette - light blue, light purple, light red
COLOR_PALETTE = {
    "Random Forest": "#A8D8EA",    # Light blue
    "XGBoost": "#E8D0F0",          # Light purple
    "LightGBM": "#F5C6C6",         # Light red
```

```
}

# Soft colors for Figure 1
FIGURE1_COLORS = ["#A8D8EA", "#E8D0F0", "#F5C6C6"]  # Light blue, light purple, light red

# Gradient soft colors for Figure 2
FIGURE2_COLORS = ["#A8D8EA", "#B8E0EA", "#E8D0F0", "#F0D8F5", "#F5C6C6", "#FAD0D0"]

# MODIFY THESE VARIABLES TO MATCH YOUR EXCEL COLUMN NAMES
FEATURES = [
    "adaptive behavior",  # Change this to match your Excel column name
    "gross motor",        # Change this to match your Excel column name
    "fine motor",         # Change this to match your Excel column name
    "language",           # Change this to match your Excel column name
    "personal-social",    # Change this to match your Excel column name
]
TARGET = "CARS scores"    # Change this to match your Excel column name


def build_parser() -> argparse.ArgumentParser:
    parser = argparse.ArgumentParser(description="Machine Learning Analysis for CARS Score Prediction")
    parser.add_argument("--data", type=Path, default=None, help="Path to the input data file")
    parser.add_argument("--seed", type=int, default=42, help="Random seed for reproducibility")
    parser.add_argument("--test-size", type=float, default=0.2, help="Proportion of test set")
    parser.add_argument("--cv-folds", type=int, default=5, help="Number of cross-validation folds")
    parser.add_argument("--output-dir", type=Path, default=Path("vitamin D total sample_outputs"), help="Output directory")
    parser.add_argument("--no-show", action="store_true", help="Do not display figures")
    return parser


def detect_data_path(manual_path: Path | None) -> Path:
    if manual_path is not None:
        if not manual_path.exists():
            raise FileNotFoundError(f"Data file not found: {manual_path}")
        return manual_path
    default_path = Path(r'C:/age at first diagnosis/vitamin/25(OH)D/paper/total sample.xlsx')
    if default_path.exists():
        return default_path
    raise FileNotFoundError("Data file not found. Please specify the path using --data argument.")
```

```python
def load_data(path: Path) -> pd.DataFrame:
    data = pd.read_excel(path)
    numeric_cols = data.select_dtypes(include=[np.number]).columns
    data[numeric_cols] = data[numeric_cols].fillna(data[numeric_cols].mean())

    # Print actual column names for debugging
    print("\nActual column names in Excel file:")
    for i, col in enumerate(data.columns):
        print(f"    {i+1}. '{col}'")
    print()

    return data


def check_required_columns(df: pd.DataFrame, columns: Iterable[str]) -> None:
    missing = [col for col in columns if col not in df.columns]
    if missing:
        print(f"\nERROR: Missing columns: {missing}")
        print("\nAvailable columns in your Excel file:")
        for col in df.columns:
            print(f"    - '{col}'")
        raise ValueError(f"Missing required columns in data: {missing}")


def make_ml_models(seed: int) -> dict[str, object]:
    models: dict[str, object] = {
        "Random Forest": RandomForestRegressor(n_estimators=400, random_state=seed),
    }
    if XGBRegressor is not None:
        models["XGBoost"] = XGBRegressor(
            n_estimators=400,
            learning_rate=0.05,
            max_depth=4,
            subsample=0.8,
            colsample_bytree=0.8,
            random_state=seed,
            objective="reg:squarederror",
        )
    if LGBMRegressor is not None:
        models["LightGBM"] = LGBMRegressor(
            n_estimators=400,
            learning_rate=0.05,
```

```
            max_depth=-1,
            num_leaves=31,
            random_state=seed,
            verbose=-1,
        )
    return models


def evaluate_ml_models(data: pd.DataFrame, seed: int, test_size: float, cv_folds: int) -> tuple[pd.DataFrame, pd.DataFrame]:
    X = data[FEATURES].copy()
    y = data[TARGET].copy()
    X_train, X_test, y_train, y_test = train_test_split(X, y, test_size=test_size, random_state=seed)
    cv = KFold(n_splits=cv_folds, shuffle=True, random_state=seed)
    models = make_ml_models(seed)
    metric_rows: list[dict[str, float | str]] = []
    importance_dict: dict[str, np.ndarray] = {"Feature": np.array(FEATURES)}

    for name, model in models.items():
        scores = cross_validate(
            model,
            X_train,
            y_train,
            cv=cv,
            scoring=("r2", "neg_root_mean_squared_error", "neg_mean_absolute_error"),
            n_jobs=1,
            error_score="raise",
        )
        model.fit(X_train, y_train)
        pred = model.predict(X_test)
        metric_rows.append(
            {
                "Model": name,
                "CV_R2_mean": float(np.mean(scores["test_r2"])),
                "CV_RMSE_mean": float(np.mean(-scores["test_neg_root_mean_squared_error"])),
                "CV_MAE_mean": float(np.mean(-scores["test_neg_mean_absolute_error"])),
                "Test_R2": float(r2_score(y_test, pred)),
                "Test_RMSE": float(np.sqrt(mean_squared_error(y_test, pred))),
                "Test_MAE": float(mean_absolute_error(y_test, pred)),
            }
        )
```

```
        if hasattr(model, "feature_importances_"):
            raw_imp = np.asarray(model.feature_importances_, dtype=float)
        else:
            perm = permutation_importance(model, X_test, y_test, n_repeats=20,
random_state=seed, scoring="r2")
            raw_imp = np.asarray(perm.importances_mean, dtype=float)

        if np.allclose(raw_imp.sum(), 0):
            norm_imp = np.zeros_like(raw_imp)
        else:
            norm_imp = 100.0 * raw_imp / raw_imp.sum()
        importance_dict[name] = norm_imp

    metric_df = pd.DataFrame(metric_rows).sort_values("Test_R2",
ascending=False).reset_index(drop=True)

    # Create feature importance DataFrame
    imp_df = pd.DataFrame(importance_dict)

    # ===== Sort by comprehensive importance across all models =====
    # Get all model column names (excluding Feature column)
    model_columns = [col for col in imp_df.columns if col != "Feature"]

    # Calculate the total importance percentage for each feature across all models
    imp_df["Total_Importance"] = imp_df[model_columns].sum(axis=1)

    # Sort by total importance percentage in descending order (most important features at top)
    imp_df = imp_df.sort_values("Total_Importance", ascending=False)

    # Remove temporary calculation column to keep DataFrame concise
    imp_df = imp_df.drop(columns=["Total_Importance"])
    # ===== End of modification =====

    return metric_df, imp_df


def plot_ml_importance(imp_df: pd.DataFrame, output_file: Path, no_show: bool) -> None:
    """Feature importance comparison across ML models - features sorted by comprehensive
importance"""

    # Use sorted DataFrame
    plot_df = imp_df.copy()
```

```
    # Get sorted feature list (already sorted by comprehensive importance descending)
    sorted_features = plot_df["Feature"].tolist()

    # Convert to long format
    melted = plot_df.melt(id_vars="Feature", var_name="Model", value_name="Importance Contribution (%)")

    # Set up figure with clean styling
    plt.figure(figsize=(12, 8), facecolor='white')
    ax = plt.gca()
    ax.set_facecolor('#F8F9FA')

    # Use soft color palette
    palette = {model: COLOR_PALETTE.get(model, "#D3D3D3") for model in melted["Model"].unique()}

    # Create bar plot with sorted features
    sns.barplot(
        data=melted,
        x="Importance Contribution (%)",
        y="Feature",
        hue="Model",
        palette=palette,
        alpha=0.85,
        edgecolor='white',
        linewidth=1,
        order=sorted_features
    )

    # Add value labels
    for container in ax.containers:
        ax.bar_label(container, fmt='%.1f%%', fontsize=9, padding=3)

    plt.title("Feature Importance Comparison Across Machine Learning Models for CARS Scores Prediction",
              fontsize=16, pad=20, fontweight='bold', color='#2C3E50')
    plt.xlabel("Feature Importance Contribution (%)", fontsize=12, fontweight='medium', color='#34495E')
    plt.ylabel("Predictor Variable", fontsize=12, fontweight='medium', color='#34495E')
    plt.grid(axis="x", linestyle="--", alpha=0.3, color='#95A5A6')
    plt.legend(title="Model", title_fontsize=11, fontsize=10, loc='best', frameon=True, fancybox=True, shadow=True)

    # Adjust layout
```

```
    plt.tight_layout()
    output_file.parent.mkdir(parents=True, exist_ok=True)
    # Save as TIFF with high quality
    plt.savefig(output_file, dpi=300, bbox_inches="tight", facecolor='white', format='tiff')
    if no_show:
        plt.close()
    else:
        plt.show()


def plot_normalized_importance(imp_df: pd.DataFrame, output_file: Path, no_show: bool) -> None:
    """Stacked bar plot of normalized feature importance across models - features sorted by comprehensive importance"""

    # Use sorted DataFrame
    plot_df = imp_df.copy()

    # Create stacked bar plot
    fig, ax = plt.subplots(figsize=(14, 8), facecolor='white')
    ax.set_facecolor('#F8F9FA')

    # Get sorted features (already sorted by comprehensive importance descending)
    features = plot_df["Feature"].values
    models = [col for col in plot_df.columns if col != "Feature"]

    # Use soft gradient color scheme
    if len(models) == 2:
        colors = ["#A8D8EA", "#F5C6C6"]  # Light blue, light red
    elif len(models) == 3:
        colors = ["#A8D8EA", "#E8D0F0", "#F5C6C6"]  # Light blue, light purple, light red
    else:
        # Use soft gradient colors
        colors = sns.color_palette(["#A8D8EA", "#B8E0EA", "#E8D0F0", "#F0D8F5", "#F5C6C6", "#FAD0D0"], n_colors=len(models))

    # Draw stacked bars with sorted features
    bottom = np.zeros(len(features))
    for idx, (model, color) in enumerate(zip(models, colors)):
        values = plot_df[model].values
        bars = ax.barh(
            features, values, left=bottom,
            label=model, color=color,
            edgecolor='white', linewidth=1.5, alpha=0.9
```

```
        )
        bottom += values

        # Add value labels on bars
        for i, (val, total) in enumerate(zip(values, bottom)):
            if val > 3:    # Only show labels for values > 3%
                # Adjust font size based on bar width
                if val > 15:
                    fontsize = 10
                elif val > 8:
                    fontsize = 9
                else:
                    fontsize = 8

                text_color = '#2C3E50'
                ax.text(total - val/2, i, f'{val:.1f}%',
                       ha='center', va='center',
                       fontsize=fontsize, fontweight='bold',
                       color=text_color)

    ax.set_xlabel('Normalized Contribution (%)', fontsize=12, fontweight='medium',
color='#34495E')
    ax.set_ylabel('Predictor Variable', fontsize=12, fontweight='medium', color='#34495E')
    ax.set_title('Stacked Comparison of Normalized Feature Importance Across Models for CARS
Scores Prediction',
                 fontsize=16, pad=20, fontweight='bold', color='#2C3E50')

    # Set legend
    ax.legend(loc='lower right', bbox_to_anchor=(1.0, 0.0),
              fontsize=10, frameon=True, fancybox=True, shadow=True,
              title="Model", title_fontsize=11)

    # Set grid
    ax.grid(axis='x', linestyle='--', alpha=0.3, color='#95A5A6')

    # Set x-axis range
    ax.set_xlim(0, 100)

    # Beautify axes
    ax.tick_params(axis='y', labelsize=11, colors='#2C3E50')
    ax.tick_params(axis='x', labelsize=10, colors='#2C3E50')

    # Invert y-axis to show most important feature at top
    ax.invert_yaxis()
```

```
    plt.tight_layout()
    output_file.parent.mkdir(parents=True, exist_ok=True)
    # Save as TIFF with high quality
    plt.savefig(output_file, dpi=300, bbox_inches="tight", facecolor='white', format='tiff')
    if no_show:
        plt.close()
    else:
        plt.show()


def run_shap_analysis(data: pd.DataFrame, seed: int, test_size: float, ml_dir: Path, no_show: bool) -> str:
    if shap is None:
        return "SHAP analysis skipped: shap package not installed."
    if XGBRegressor is None:
        return "SHAP analysis skipped: xgboost package not installed."

    X = data[FEATURES].copy()
    y = data[TARGET].copy()
    X_train, X_test, y_train, _ = train_test_split(X, y, test_size=test_size, random_state=seed)

    model = XGBRegressor(
        n_estimators=400,
        learning_rate=0.05,
        max_depth=4,
        subsample=0.8,
        colsample_bytree=0.8,
        random_state=seed,
        objective="reg:squarederror",
    )
    model.fit(X_train, y_train)

    explainer = shap.TreeExplainer(model)
    shap_values = explainer.shap_values(X_test)
    shap_matrix = np.asarray(shap_values)
    if shap_matrix.ndim == 3:
        shap_matrix = shap_matrix[0]

    mean_abs_shap = np.abs(shap_matrix).mean(axis=0)

    # Create SHAP table sorted by importance
    shap_table = pd.DataFrame({"Feature": FEATURES, "Mean_Absolute_SHAP": mean_abs_shap})
```

```python
    shap_table = shap_table.sort_values("Mean_Absolute_SHAP", ascending=False)
    shap_table.to_csv(ml_dir / "vitamin D total sample_shap_mean_abs.csv", index=False, encoding="utf-8-sig")

    plt.figure(figsize=(10, 7), facecolor='white')
    # Summary plot automatically sorts by importance
    shap.summary_plot(shap_values, X_test, plot_type="dot", show=False, color=plt.cm.RdYlBu)
    plt.title("SHAP Feature Importance: Impact on CARS Scores Prediction", fontsize=14, pad=16, fontweight='bold')
    plt.xlabel("SHAP Value (Impact on Model Output)", fontsize=11)
    plt.tight_layout()
    # Save as TIFF with high quality
    plt.savefig(ml_dir / "vitamin D total sample_shap_summary.tiff", dpi=300, bbox_inches="tight", facecolor='white', format='tiff')
    if no_show:
        plt.close()
    else:
        plt.show()

    return "SHAP summary plot and table generated successfully."


def pretty(df: pd.DataFrame) -> str:
    return df.to_string(index=False, float_format=lambda x: f"{x:.6f}")


def run_ml_track(data: pd.DataFrame, seed: int, test_size: float, cv_folds: int, output_dir: Path, no_show: bool) -> None:
    metric_df, imp_df = evaluate_ml_models(data, seed=seed, test_size=test_size, cv_folds=cv_folds)
    ml_dir = output_dir / "ml"
    ml_dir.mkdir(parents=True, exist_ok=True)

    # Save CSV files with new naming convention
    metric_df.to_csv(ml_dir / "vitamin D total sample_model_metrics.csv", index=False, encoding="utf-8-sig")
    imp_df.to_csv(ml_dir / "vitamin D total sample_feature_importance.csv", index=False, encoding="utf-8-sig")

    # Figure 1: Feature importance comparison (sorted by total importance descending)
    plot_ml_importance(imp_df, ml_dir / "vitamin D total sample_feature_importance.tiff", no_show=no_show)
```

```
    # Figure 2: Stacked normalized importance (sorted by total importance descending)
    plot_normalized_importance(imp_df, ml_dir / "vitamin D total sample_normalized_importance.tiff", no_show=no_show)

    shap_status = run_shap_analysis(data=data, seed=seed, test_size=test_size, ml_dir=ml_dir, no_show=no_show)

    print("\n" + "="*50)
    print("MACHINE LEARNING ANALYSIS RESULTS")
    print("="*50)
    print("\n[Model Performance Metrics]")
    print(pretty(metric_df))
    print("\n[Feature Importance (%)]")
    print(pretty(imp_df))

    if (ml_dir / "vitamin D total sample_shap_mean_abs.csv").exists():
        shap_df = pd.read_csv(ml_dir / "vitamin D total sample_shap_mean_abs.csv")
        print("\n[SHAP Mean Absolute Contribution]")
        print(pretty(shap_df))

    print(f"\n[SHAP Analysis] {shap_status}")
    print(f"\nOutput Directory: {ml_dir}")
    print(f"\nGenerated Figures:")
    print(f"    - Feature importance comparison → {ml_dir / 'vitamin D total sample_feature_importance.tiff'}")
    print(f"    - Stacked normalized importance → {ml_dir / 'vitamin D total sample_normalized_importance.tiff'}")
    print(f"    - SHAP summary plot → {ml_dir / 'vitamin D total sample_shap_summary.tiff'}")


def main() -> None:
    args = build_parser().parse_args()
    path = detect_data_path(args.data)
    data = load_data(path)
    check_required_columns(data, FEATURES + [TARGET])
    print(f"Data file: {path}")
    print(f"Sample size: {len(data)}")
    print(f"Output directory: {args.output_dir}")
    run_ml_track(data, seed=args.seed, test_size=args.test_size, cv_folds=args.cv_folds, output_dir=args.output_dir, no_show=args.no_show)


if __name__ == "__main__":
    main()
```

**Machine learning feature importance ranking code for the normal vitamin D (NVD) group**

```
# -*- coding: utf-8 -*-
"""
Created on Sat Apr 18 04:20:37 2026

"""

import argparse
from pathlib import Path
from typing import Iterable

import matplotlib.pyplot as plt
import numpy as np
import pandas as pd
import seaborn as sns
from sklearn.ensemble import RandomForestRegressor
from sklearn.inspection import permutation_importance
from sklearn.metrics import mean_absolute_error, mean_squared_error, r2_score
from sklearn.model_selection import KFold, cross_validate, train_test_split

try:
    from xgboost import XGBRegressor
except Exception:
    XGBRegressor = None

try:
    from lightgbm import LGBMRegressor
except Exception:
    LGBMRegressor = None

try:
    import shap
except Exception:
    shap = None


plt.rcParams["font.sans-serif"] = ["Arial"]
plt.rcParams["axes.unicode_minus"] = False

# Soft color palette - light blue, light purple, light red
COLOR_PALETTE = {
    "Random Forest": "#A8D8EA",    # Light blue
    "XGBoost": "#E8D0F0",              # Light purple
```

```
        "LightGBM": "#F5C6C6",          # Light red
    }

    # Soft colors for Figure 1
    FIGURE1_COLORS = ["#A8D8EA", "#E8D0F0", "#F5C6C6"]    # Light blue, light purple, light
red

    # Gradient soft colors for Figure 2
    FIGURE2_COLORS = ["#A8D8EA", "#B8E0EA", "#E8D0F0", "#F0D8F5", "#F5C6C6",
"#FAD0D0"]

    # MODIFY THESE VARIABLES TO MATCH YOUR EXCEL COLUMN NAMES
    FEATURES = [
        "adaptive behavior",    # Change this to match your Excel column name
        "gross motor",          # Change this to match your Excel column name
        "fine motor",           # Change this to match your Excel column name
        "language",             # Change this to match your Excel column name
        "personal-social",      # Change this to match your Excel column name
    ]
    TARGET = "CARS scores"      # Change this to match your Excel column name


    def build_parser() -> argparse.ArgumentParser:
        parser = argparse.ArgumentParser(description="Machine Learning Analysis for CARS
Score Prediction")
        parser.add_argument("--data", type=Path, default=None, help="Path to the input data
file")
        parser.add_argument("--seed", type=int, default=42, help="Random seed for
reproducibility")
        parser.add_argument("--test-size", type=float, default=0.2, help="Proportion of test
set")
        parser.add_argument("--cv-folds", type=int, default=5, help="Number of
cross-validation folds")
        parser.add_argument("--output-dir", type=Path, default=Path("normal vitamin D
(NVD)_outputs"), help="Output directory")
        parser.add_argument("--no-show", action="store_true", help="Do not display figures")
        return parser


    def detect_data_path(manual_path: Path | None) -> Path:
        if manual_path is not None:
            if not manual_path.exists():
                raise FileNotFoundError(f"Data file not found: {manual_path}")
            return manual_path
```

```
    default_path = Path(r'C:/age at first diagnosis/vitamin/25(OH)D/paper/normal vitamin D (NVD).xlsx')
    if default_path.exists():
        return default_path
    raise FileNotFoundError("Data file not found. Please specify the path using --data argument.")


def load_data(path: Path) -> pd.DataFrame:
    data = pd.read_excel(path)
    numeric_cols = data.select_dtypes(include=[np.number]).columns
    data[numeric_cols] = data[numeric_cols].fillna(data[numeric_cols].mean())

    # Print actual column names for debugging
    print("\nActual column names in Excel file:")
    for i, col in enumerate(data.columns):
        print(f"    {i+1}. '{col}'")
    print()

    return data


def check_required_columns(df: pd.DataFrame, columns: Iterable[str]) -> None:
    missing = [col for col in columns if col not in df.columns]
    if missing:
        print(f"\nERROR: Missing columns: {missing}")
        print("\nAvailable columns in your Excel file:")
        for col in df.columns:
            print(f"    - '{col}'")
        raise ValueError(f"Missing required columns in data: {missing}")


def make_ml_models(seed: int) -> dict[str, object]:
    models: dict[str, object] = {
        "Random Forest": RandomForestRegressor(n_estimators=400, random_state=seed),
    }
    if XGBRegressor is not None:
        models["XGBoost"] = XGBRegressor(
            n_estimators=400,
            learning_rate=0.05,
            max_depth=4,
            subsample=0.8,
            colsample_bytree=0.8,
```

```
            random_state=seed,
            objective="reg:squarederror",
        )
    if LGBMRegressor is not None:
        models["LightGBM"] = LGBMRegressor(
            n_estimators=400,
            learning_rate=0.05,
            max_depth=-1,
            num_leaves=31,
            random_state=seed,
            verbose=-1,
        )
    return models


def evaluate_ml_models(data: pd.DataFrame, seed: int, test_size: float, cv_folds: int) -> tuple[pd.DataFrame, pd.DataFrame]:
    X = data[FEATURES].copy()
    y = data[TARGET].copy()
    X_train, X_test, y_train, y_test = train_test_split(X, y, test_size=test_size, random_state=seed)
    cv = KFold(n_splits=cv_folds, shuffle=True, random_state=seed)
    models = make_ml_models(seed)
    metric_rows: list[dict[str, float | str]] = []
    importance_dict: dict[str, np.ndarray] = {"Feature": np.array(FEATURES)}

    for name, model in models.items():
        scores = cross_validate(
            model,
            X_train,
            y_train,
            cv=cv,
            scoring=("r2", "neg_root_mean_squared_error", "neg_mean_absolute_error"),
            n_jobs=1,
            error_score="raise",
        )
        model.fit(X_train, y_train)
        pred = model.predict(X_test)
        metric_rows.append(
            {
                "Model": name,
                "CV_R2_mean": float(np.mean(scores["test_r2"])),
                "CV_RMSE_mean":
```

```
float(np.mean(-scores["test_neg_root_mean_squared_error"])),
                    "CV_MAE_mean":
float(np.mean(-scores["test_neg_mean_absolute_error"])),
                    "Test_R2": float(r2_score(y_test, pred)),
                    "Test_RMSE": float(np.sqrt(mean_squared_error(y_test, pred))),
                    "Test_MAE": float(mean_absolute_error(y_test, pred)),
                }
            )

            if hasattr(model, "feature_importances_"):
                raw_imp = np.asarray(model.feature_importances_, dtype=float)
            else:
                perm = permutation_importance(model, X_test, y_test, n_repeats=20,
random_state=seed, scoring="r2")
                raw_imp = np.asarray(perm.importances_mean, dtype=float)

            if np.allclose(raw_imp.sum(), 0):
                norm_imp = np.zeros_like(raw_imp)
            else:
                norm_imp = 100.0 * raw_imp / raw_imp.sum()
            importance_dict[name] = norm_imp

        metric_df = pd.DataFrame(metric_rows).sort_values("Test_R2",
ascending=False).reset_index(drop=True)

        # Create feature importance DataFrame
        imp_df = pd.DataFrame(importance_dict)

        # ===== Sort by comprehensive importance across all models =====
        # Get all model column names (excluding Feature column)
        model_columns = [col for col in imp_df.columns if col != "Feature"]

        # Calculate the total importance percentage for each feature across all models
        imp_df["Total_Importance"] = imp_df[model_columns].sum(axis=1)

        # Sort by total importance percentage in descending order (most important features at
top)
        imp_df = imp_df.sort_values("Total_Importance", ascending=False)

        # Remove temporary calculation column to keep DataFrame concise
        imp_df = imp_df.drop(columns=["Total_Importance"])
        # ===== End of modification =====

        return metric_df, imp_df
```

```
def plot_ml_importance(imp_df: pd.DataFrame, output_file: Path, no_show: bool) -> None:
    """Feature importance comparison across ML models - features sorted by comprehensive importance"""

    # Use sorted DataFrame
    plot_df = imp_df.copy()

    # Get sorted feature list (already sorted by total importance descending)
    sorted_features = plot_df["Feature"].tolist()

    # Convert to long format
    melted = plot_df.melt(id_vars="Feature", var_name="Model", value_name="Importance Contribution (%)")

    # Set up figure with clean styling
    plt.figure(figsize=(12, 8), facecolor='white')
    ax = plt.gca()
    ax.set_facecolor('#F8F9FA')

    # Use soft color palette
    palette = {model: COLOR_PALETTE.get(model, "#D3D3D3") for model in melted["Model"].unique()}

    # Create bar plot with sorted features
    sns.barplot(
        data=melted,
        x="Importance Contribution (%)",
        y="Feature",
        hue="Model",
        palette=palette,
        alpha=0.85,
        edgecolor='white',
        linewidth=1,
        order=sorted_features
    )

    # Add value labels
    for container in ax.containers:
        ax.bar_label(container, fmt='%.1f%%', fontsize=9, padding=3)

    plt.title("Feature Importance Comparison Across Machine Learning Models for CARS Scores Prediction",
```

```
                    fontsize=16, pad=20, fontweight='bold', color='#2C3E50')
        plt.xlabel("Feature Importance Contribution (%)", fontsize=12, fontweight='medium',
color='#34495E')
        plt.ylabel("Predictor Variable", fontsize=12, fontweight='medium', color='#34495E')
        plt.grid(axis="x", linestyle="--", alpha=0.3, color='#95A5A6')
        plt.legend(title="Model", title_fontsize=11, fontsize=10, loc='best', frameon=True,
fancybox=True, shadow=True)

        # Adjust layout
        plt.tight_layout()
        output_file.parent.mkdir(parents=True, exist_ok=True)
        # Save as TIFF with high quality
        plt.savefig(output_file, dpi=300, bbox_inches="tight", facecolor='white', format='tiff')
        if no_show:
            plt.close()
        else:
            plt.show()


    def plot_normalized_importance(imp_df: pd.DataFrame, output_file: Path, no_show: bool)
-> None:
        """Stacked bar plot of normalized feature importance across models - features sorted
by comprehensive importance"""

        # Use sorted DataFrame
        plot_df = imp_df.copy()

        # Create stacked bar plot
        fig, ax = plt.subplots(figsize=(14, 8), facecolor='white')
        ax.set_facecolor('#F8F9FA')

        # Get sorted features (already sorted by total importance descending)
        features = plot_df["Feature"].values
        models = [col for col in plot_df.columns if col != "Feature"]

        # Use soft gradient color scheme
        if len(models) == 2:
            colors = ["#A8D8EA", "#F5C6C6"]    # Light blue, light red
        elif len(models) == 3:
            colors = ["#A8D8EA", "#E8D0F0", "#F5C6C6"]    # Light blue, light purple, light red
        else:
            # Use soft gradient colors
            colors = sns.color_palette(["#A8D8EA", "#B8E0EA", "#E8D0F0", "#F0D8F5",
"#F5C6C6", "#FAD0D0"], n_colors=len(models))
```

```
    # Draw stacked bars with sorted features
    bottom = np.zeros(len(features))
    for idx, (model, color) in enumerate(zip(models, colors)):
        values = plot_df[model].values
        bars = ax.barh(
            features, values, left=bottom,
            label=model, color=color,
            edgecolor='white', linewidth=1.5, alpha=0.9
        )
        bottom += values

        # Add value labels on bars
        for i, (val, total) in enumerate(zip(values, bottom)):
            if val > 3:    # Only show labels for values > 3%
                # Adjust font size based on bar width
                if val > 15:
                    fontsize = 10
                elif val > 8:
                    fontsize = 9
                else:
                    fontsize = 8

                text_color = '#2C3E50'
                ax.text(total - val/2, i, f'{val:.1f}%',
                       ha='center', va='center',
                       fontsize=fontsize, fontweight='bold',
                       color=text_color)

    ax.set_xlabel('Normalized Contribution (%)', fontsize=12, fontweight='medium',
color='#34495E')
    ax.set_ylabel('Predictor Variable', fontsize=12, fontweight='medium', color='#34495E')
    ax.set_title('(a)',
                 fontsize=20, pad=20, fontweight='bold', color='#2C3E50')

    # Set legend
    ax.legend(loc='lower right', bbox_to_anchor=(1.0, 0.0),
              fontsize=10, frameon=True, fancybox=True, shadow=True,
              title="Model", title_fontsize=11)

    # Set grid
    ax.grid(axis='x', linestyle='--', alpha=0.3, color='#95A5A6')

    # Set x-axis range
```

```python
        ax.set_xlim(0, 100)

        # Beautify axes
        ax.tick_params(axis='y', labelsize=11, colors='#2C3E50')
        ax.tick_params(axis='x', labelsize=10, colors='#2C3E50')

        # Invert y-axis to show most important feature at top
        ax.invert_yaxis()

        plt.tight_layout()
        output_file.parent.mkdir(parents=True, exist_ok=True)
        # Save as TIFF with high quality
        plt.savefig(output_file, dpi=300, bbox_inches="tight", facecolor='white', format='tiff')
        if no_show:
            plt.close()
        else:
            plt.show()


    def run_shap_analysis(data: pd.DataFrame, seed: int, test_size: float, ml_dir: Path, no_show:
bool) -> str:
        if shap is None:
            return "SHAP analysis skipped: shap package not installed."
        if XGBRegressor is None:
            return "SHAP analysis skipped: xgboost package not installed."

        X = data[FEATURES].copy()
        y = data[TARGET].copy()
        X_train, X_test, y_train, _ = train_test_split(X, y, test_size=test_size,
random_state=seed)

        model = XGBRegressor(
            n_estimators=400,
            learning_rate=0.05,
            max_depth=4,
            subsample=0.8,
            colsample_bytree=0.8,
            random_state=seed,
            objective="reg:squarederror",
        )
        model.fit(X_train, y_train)

        explainer = shap.TreeExplainer(model)
        shap_values = explainer.shap_values(X_test)
```

```
        shap_matrix = np.asarray(shap_values)
        if shap_matrix.ndim == 3:
            shap_matrix = shap_matrix[0]

        mean_abs_shap = np.abs(shap_matrix).mean(axis=0)

        # Create SHAP table sorted by importance
        shap_table = pd.DataFrame({"Feature": FEATURES, "Mean_Absolute_SHAP": mean_abs_shap})
        shap_table = shap_table.sort_values("Mean_Absolute_SHAP", ascending=False)
        shap_table.to_csv(ml_dir / "normal vitamin D (NVD)_shap_mean_abs.csv", index=False, encoding="utf-8-sig")

        plt.figure(figsize=(10, 7), facecolor='white')
        # Summary plot automatically sorts by importance
        shap.summary_plot(shap_values, X_test, plot_type="dot", show=False, color=plt.cm.RdYlBu)
        plt.title("SHAP Feature Importance: Impact on CARS Scores Prediction", fontsize=14, pad=16, fontweight='bold')
        plt.xlabel("SHAP Value (Impact on Model Output)", fontsize=11)
        plt.tight_layout()
        # Save as TIFF with high quality
        plt.savefig(ml_dir / "normal vitamin D (NVD)_shap_summary.tiff", dpi=300, bbox_inches="tight", facecolor='white', format='tiff')
        if no_show:
            plt.close()
        else:
            plt.show()

        return "SHAP summary plot and table generated successfully."


    def pretty(df: pd.DataFrame) -> str:
        return df.to_string(index=False, float_format=lambda x: f"{x:.6f}")


    def run_ml_track(data: pd.DataFrame, seed: int, test_size: float, cv_folds: int, output_dir: Path, no_show: bool) -> None:
        metric_df, imp_df = evaluate_ml_models(data, seed=seed, test_size=test_size, cv_folds=cv_folds)
        ml_dir = output_dir / "ml"
        ml_dir.mkdir(parents=True, exist_ok=True)

        # Save CSV files with new naming convention
```

```
        metric_df.to_csv(ml_dir / "normal vitamin D (NVD)_model_metrics.csv", index=False, encoding="utf-8-sig")
        imp_df.to_csv(ml_dir / "normal vitamin D (NVD)_feature_importance.csv", index=False, encoding="utf-8-sig")

        # Figure 1: Feature importance comparison (sorted by total importance descending)
        plot_ml_importance(imp_df, ml_dir / "normal vitamin D (NVD)_feature_importance.tiff", no_show=no_show)

        # Figure 2: Stacked normalized importance (sorted by total importance descending)
        plot_normalized_importance(imp_df, ml_dir / "normal vitamin D (NVD)_normalized_importance.tiff", no_show=no_show)

        shap_status = run_shap_analysis(data=data, seed=seed, test_size=test_size, ml_dir=ml_dir, no_show=no_show)

        print("\n" + "="*50)
        print("MACHINE LEARNING ANALYSIS RESULTS")
        print("="*50)
        print("\n[Model Performance Metrics]")
        print(pretty(metric_df))
        print("\n[Feature Importance (%)]")
        print(pretty(imp_df))

        if (ml_dir / "normal vitamin D (NVD)_shap_mean_abs.csv").exists():
            shap_df = pd.read_csv(ml_dir / "normal vitamin D (NVD)_shap_mean_abs.csv")
            print("\n[SHAP Mean Absolute Contribution]")
            print(pretty(shap_df))

        print(f"\n[SHAP Analysis] {shap_status}")
        print(f"\nOutput Directory: {ml_dir}")
        print(f"\nGenerated Figures:")
        print(f"    - Feature importance comparison → {ml_dir / 'normal vitamin D (NVD)_feature_importance.tiff'}")
        print(f"    - Stacked normalized importance → {ml_dir / 'normal vitamin D (NVD)_normalized_importance.tiff'}")
        print(f"    - SHAP summary plot → {ml_dir / 'normal vitamin D (NVD)_shap_summary.tiff'}")


    def main() -> None:
        args = build_parser().parse_args()
        path = detect_data_path(args.data)
        data = load_data(path)
```

```
    check_required_columns(data, FEATURES + [TARGET])
    print(f"Data file: {path}")
    print(f"Sample size: {len(data)}")
    print(f"Output directory: {args.output_dir}")
    run_ml_track(data, seed=args.seed, test_size=args.test_size, cv_folds=args.cv_folds, output_dir=args.output_dir, no_show=args.no_show)


if __name__ == "__main__":
    main()
```

**Machine learning feature importance ranking code for the vitamin D insufficiency (VDI) group.**

```
# -*- coding: utf-8 -*-
"""
Created on Sat Apr 18 04:20:37 2026

"""

import argparse
from pathlib import Path
from typing import Iterable

import matplotlib.pyplot as plt
import numpy as np
import pandas as pd
import seaborn as sns
from sklearn.ensemble import RandomForestRegressor
from sklearn.inspection import permutation_importance
from sklearn.metrics import mean_absolute_error, mean_squared_error, r2_score
from sklearn.model_selection import KFold, cross_validate, train_test_split

try:
    from xgboost import XGBRegressor
except Exception:
    XGBRegressor = None

try:
    from lightgbm import LGBMRegressor
except Exception:
    LGBMRegressor = None

try:
    import shap
except Exception:
    shap = None


plt.rcParams["font.sans-serif"] = ["Arial"]
plt.rcParams["axes.unicode_minus"] = False

# Soft color palette - light blue, light purple, light red
COLOR_PALETTE = {
    "Random Forest": "#A8D8EA",    # Light blue
    "XGBoost": "#E8D0F0",              # Light purple
```

```
        "LightGBM": "#F5C6C6",              # Light red
    }

    # Soft colors for Figure 1
    FIGURE1_COLORS = ["#A8D8EA", "#E8D0F0", "#F5C6C6"]    # Light blue, light purple, light
red

    # Gradient soft colors for Figure 2
    FIGURE2_COLORS = ["#A8D8EA", "#B8E0EA", "#E8D0F0", "#F0D8F5", "#F5C6C6",
"#FAD0D0"]

    # MODIFY THESE VARIABLES TO MATCH YOUR EXCEL COLUMN NAMES
    FEATURES = [
        "adaptive behavior",    # Change this to match your Excel column name
        "gross motor",              # Change this to match your Excel column name
        "fine motor",                # Change this to match your Excel column name
        "language",                    # Change this to match your Excel column name
        "personal-social",       # Change this to match your Excel column name
    ]
    TARGET = "CARS scores"       # Change this to match your Excel column name


    def build_parser() -> argparse.ArgumentParser:
        parser = argparse.ArgumentParser(description="Machine Learning Analysis for CARS
Score Prediction")
        parser.add_argument("--data", type=Path, default=None, help="Path to the input data
file")
        parser.add_argument("--seed", type=int, default=42, help="Random seed for
reproducibility")
        parser.add_argument("--test-size", type=float, default=0.2, help="Proportion of test
set")
        parser.add_argument("--cv-folds", type=int, default=5, help="Number of
cross-validation folds")
        parser.add_argument("--output-dir", type=Path, default=Path("vitamin D insufficiency
(VDI)_outputs"), help="Output directory")
        parser.add_argument("--no-show", action="store_true", help="Do not display figures")
        return parser


    def detect_data_path(manual_path: Path | None) -> Path:
        if manual_path is not None:
            if not manual_path.exists():
                raise FileNotFoundError(f"Data file not found: {manual_path}")
            return manual_path
```

```
        default_path = Path(r'C:/age at first diagnosis/vitamin/25(OH)D/paper/vitamin D
insufficiency (VDI).xlsx')
        if default_path.exists():
            return default_path
        raise FileNotFoundError("Data file not found. Please specify the path using --data
argument.")


    def load_data(path: Path) -> pd.DataFrame:
        data = pd.read_excel(path)
        numeric_cols = data.select_dtypes(include=[np.number]).columns
        data[numeric_cols] = data[numeric_cols].fillna(data[numeric_cols].mean())

        # Print actual column names for debugging
        print("\nActual column names in Excel file:")
        for i, col in enumerate(data.columns):
            print(f"    {i+1}. '{col}'")
        print()

        return data


    def check_required_columns(df: pd.DataFrame, columns: Iterable[str]) -> None:
        missing = [col for col in columns if col not in df.columns]
        if missing:
            print(f"\nERROR: Missing columns: {missing}")
            print("\nAvailable columns in your Excel file:")
            for col in df.columns:
                print(f"    - '{col}'")
            raise ValueError(f"Missing required columns in data: {missing}")


    def make_ml_models(seed: int) -> dict[str, object]:
        models: dict[str, object] = {
            "Random Forest": RandomForestRegressor(n_estimators=400,
random_state=seed),
        }
        if XGBRegressor is not None:
            models["XGBoost"] = XGBRegressor(
                n_estimators=400,
                learning_rate=0.05,
                max_depth=4,
                subsample=0.8,
                colsample_bytree=0.8,
```

```
                random_state=seed,
                objective="reg:squarederror",
            )
        if LGBMRegressor is not None:
            models["LightGBM"] = LGBMRegressor(
                n_estimators=400,
                learning_rate=0.05,
                max_depth=-1,
                num_leaves=31,
                random_state=seed,
                verbose=-1,
            )
        return models


    def evaluate_ml_models(data: pd.DataFrame, seed: int, test_size: float, cv_folds: int) ->
tuple[pd.DataFrame, pd.DataFrame]:
        X = data[FEATURES].copy()
        y = data[TARGET].copy()
        X_train, X_test, y_train, y_test = train_test_split(X, y, test_size=test_size,
random_state=seed)
        cv = KFold(n_splits=cv_folds, shuffle=True, random_state=seed)
        models = make_ml_models(seed)
        metric_rows: list[dict[str, float | str]] = []
        importance_dict: dict[str, np.ndarray] = {"Feature": np.array(FEATURES)}

        for name, model in models.items():
            scores = cross_validate(
                model,
                X_train,
                y_train,
                cv=cv,
                scoring=("r2",                    "neg_root_mean_squared_error",
"neg_mean_absolute_error"),
                n_jobs=1,
                error_score="raise",
            )
            model.fit(X_train, y_train)
            pred = model.predict(X_test)
            metric_rows.append(
                {
                    "Model": name,
                    "CV_R2_mean": float(np.mean(scores["test_r2"])),
                    "CV_RMSE_mean":
```

```
float(np.mean(-scores["test_neg_root_mean_squared_error"])),
                    "CV_MAE_mean":
float(np.mean(-scores["test_neg_mean_absolute_error"])),
                    "Test_R2": float(r2_score(y_test, pred)),
                    "Test_RMSE": float(np.sqrt(mean_squared_error(y_test, pred))),
                    "Test_MAE": float(mean_absolute_error(y_test, pred)),
                }
            )

            if hasattr(model, "feature_importances_"):
                raw_imp = np.asarray(model.feature_importances_, dtype=float)
            else:
                perm = permutation_importance(model, X_test, y_test, n_repeats=20,
random_state=seed, scoring="r2")
                raw_imp = np.asarray(perm.importances_mean, dtype=float)

            if np.allclose(raw_imp.sum(), 0):
                norm_imp = np.zeros_like(raw_imp)
            else:
                norm_imp = 100.0 * raw_imp / raw_imp.sum()
            importance_dict[name] = norm_imp

        metric_df = pd.DataFrame(metric_rows).sort_values("Test_R2",
ascending=False).reset_index(drop=True)

        # Create feature importance DataFrame
        imp_df = pd.DataFrame(importance_dict)

        # ===== Sort by comprehensive importance across all models =====
        # Calculate comprehensive importance (sum of importance across all models)
        model_columns = [col for col in imp_df.columns if col != "Feature"]
        imp_df["Total_Importance"] = imp_df[model_columns].sum(axis=1)

        # Sort by comprehensive importance in descending order
        imp_df = imp_df.sort_values("Total_Importance", ascending=False)

        # Remove temporary column
        imp_df = imp_df.drop(columns=["Total_Importance"])
        # ===== End of modification =====

        return metric_df, imp_df


    def plot_ml_importance(imp_df: pd.DataFrame, output_file: Path, no_show: bool) -> None:
```

```
"""Feature importance comparison across ML models - features sorted by comprehensive importance"""

# Use sorted DataFrame
plot_df = imp_df.copy()

# Get sorted feature list (already sorted by comprehensive importance descending)
sorted_features = plot_df["Feature"].tolist()

# Convert to long format
melted = plot_df.melt(id_vars="Feature", var_name="Model", value_name="Importance Contribution (%)")

# Set up figure with clean styling
plt.figure(figsize=(12, 8), facecolor='white')
ax = plt.gca()
ax.set_facecolor('#F8F9FA')

# Use soft color palette
palette = {model: COLOR_PALETTE.get(model, "#D3D3D3") for model in melted["Model"].unique()}

# Create bar plot with sorted features
sns.barplot(
    data=melted,
    x="Importance Contribution (%)",
    y="Feature",
    hue="Model",
    palette=palette,
    alpha=0.85,
    edgecolor='white',
    linewidth=1,
    order=sorted_features
)

# Add value labels
for container in ax.containers:
    ax.bar_label(container, fmt='%.1f%%', fontsize=9, padding=3)

plt.title("Feature Importance Comparison Across Machine Learning Models for CARS Scores Prediction",
          fontsize=16, pad=20, fontweight='bold', color='#2C3E50')
plt.xlabel("Feature Importance Contribution (%)", fontsize=12, fontweight='medium', color='#34495E')
```

```
        plt.ylabel("Predictor Variable", fontsize=12, fontweight='medium', color='#34495E')
        plt.grid(axis="x", linestyle="--", alpha=0.3, color='#95A5A6')
        plt.legend(title="Model", title_fontsize=11, fontsize=10, loc='best', frameon=True,
fancybox=True, shadow=True)

        # Adjust layout
        plt.tight_layout()
        output_file.parent.mkdir(parents=True, exist_ok=True)
        # Save as TIFF with high quality
        plt.savefig(output_file, dpi=300, bbox_inches="tight", facecolor='white', format='tiff')
        if no_show:
            plt.close()
        else:
            plt.show()


    def plot_normalized_importance(imp_df: pd.DataFrame, output_file: Path, no_show: bool)
-> None:
        """Stacked bar plot of normalized feature importance across models - features sorted
by comprehensive importance"""

        # Use sorted DataFrame
        plot_df = imp_df.copy()

        # Create stacked bar plot
        fig, ax = plt.subplots(figsize=(14, 8), facecolor='white')
        ax.set_facecolor('#F8F9FA')

        # Get sorted features (already sorted by comprehensive importance descending)
        features = plot_df["Feature"].values
        models = [col for col in plot_df.columns if col != "Feature"]

        # Use soft gradient color scheme
        if len(models) == 2:
            colors = ["#A8D8EA", "#F5C6C6"]  # Light blue, light red
        elif len(models) == 3:
            colors = ["#A8D8EA", "#E8D0F0", "#F5C6C6"]  # Light blue, light purple, light red
        else:
            # Use soft gradient colors
            colors = sns.color_palette(["#A8D8EA", "#B8E0EA", "#E8D0F0", "#F0D8F5",
"#F5C6C6", "#FAD0D0"], n_colors=len(models))

        # Draw stacked bars with sorted features
        bottom = np.zeros(len(features))
```

```
    for idx, (model, color) in enumerate(zip(models, colors)):
        values = plot_df[model].values
        bars = ax.barh(
            features, values, left=bottom,
            label=model, color=color,
            edgecolor='white', linewidth=1.5, alpha=0.9
        )
        bottom += values

        # Add value labels on bars
        for i, (val, total) in enumerate(zip(values, bottom)):
            if val > 3:    # Only show labels for values > 3%
                # Adjust font size based on bar width
                if val > 15:
                    fontsize = 10
                elif val > 8:
                    fontsize = 9
                else:
                    fontsize = 8

                text_color = '#2C3E50'
                ax.text(total - val/2, i, f'{val:.1f}%',
                       ha='center', va='center',
                       fontsize=fontsize, fontweight='bold',
                       color=text_color)

    ax.set_xlabel('Normalized Contribution (%)', fontsize=12, fontweight='medium',
color='#34495E')
    ax.set_ylabel('Predictor Variable', fontsize=12, fontweight='medium', color='#34495E')
    ax.set_title('(b)',
                 fontsize=20, pad=20, fontweight='bold', color='#2C3E50')

    # Set legend
    ax.legend(loc='lower right', bbox_to_anchor=(1.0, 0.0),
              fontsize=10, frameon=True, fancybox=True, shadow=True,
              title="Model", title_fontsize=11)

    # Set grid
    ax.grid(axis='x', linestyle='--', alpha=0.3, color='#95A5A6')

    # Set x-axis range
    ax.set_xlim(0, 100)

    # Beautify axes
```

```
    ax.tick_params(axis='y', labelsize=11, colors='#2C3E50')
    ax.tick_params(axis='x', labelsize=10, colors='#2C3E50')

    # Invert y-axis to show most important feature at top
    ax.invert_yaxis()

    plt.tight_layout()
    output_file.parent.mkdir(parents=True, exist_ok=True)
    # Save as TIFF with high quality
    plt.savefig(output_file, dpi=300, bbox_inches="tight", facecolor='white', format='tiff')
    if no_show:
        plt.close()
    else:
        plt.show()


def run_shap_analysis(data: pd.DataFrame, seed: int, test_size: float, ml_dir: Path, no_show: bool) -> str:
    if shap is None:
        return "SHAP analysis skipped: shap package not installed."
    if XGBRegressor is None:
        return "SHAP analysis skipped: xgboost package not installed."

    X = data[FEATURES].copy()
    y = data[TARGET].copy()
    X_train, X_test, y_train, _ = train_test_split(X, y, test_size=test_size, random_state=seed)

    model = XGBRegressor(
        n_estimators=400,
        learning_rate=0.05,
        max_depth=4,
        subsample=0.8,
        colsample_bytree=0.8,
        random_state=seed,
        objective="reg:squarederror",
    )
    model.fit(X_train, y_train)

    explainer = shap.TreeExplainer(model)
    shap_values = explainer.shap_values(X_test)
    shap_matrix = np.asarray(shap_values)
    if shap_matrix.ndim == 3:
        shap_matrix = shap_matrix[0]
```

```
        mean_abs_shap = np.abs(shap_matrix).mean(axis=0)

        # Create SHAP table sorted by importance
        shap_table = pd.DataFrame({"Feature": FEATURES, "Mean_Absolute_SHAP":
mean_abs_shap})
        shap_table = shap_table.sort_values("Mean_Absolute_SHAP", ascending=False)
        shap_table.to_csv(ml_dir / "vitamin D insufficiency (VDI)_shap_mean_abs.csv",
index=False, encoding="utf-8-sig")

        plt.figure(figsize=(10, 7), facecolor='white')
        # Summary plot automatically sorts by importance
        shap.summary_plot(shap_values, X_test, plot_type="dot", show=False,
color=plt.cm.RdYlBu)
        plt.title("SHAP Feature Importance: Impact on CARS Scores Prediction", fontsize=14,
pad=16, fontweight='bold')
        plt.xlabel("SHAP Value (Impact on Model Output)", fontsize=11)
        plt.tight_layout()
        # Save as TIFF with high quality
        plt.savefig(ml_dir / "vitamin D insufficiency (VDI)_shap_summary.tiff", dpi=300,
bbox_inches="tight", facecolor='white', format='tiff')
        if no_show:
            plt.close()
        else:
            plt.show()

        return "SHAP summary plot and table generated successfully."


    def pretty(df: pd.DataFrame) -> str:
        return df.to_string(index=False, float_format=lambda x: f"{x:.6f}")


    def run_ml_track(data: pd.DataFrame, seed: int, test_size: float, cv_folds: int, output_dir:
Path, no_show: bool) -> None:
        metric_df, imp_df = evaluate_ml_models(data, seed=seed, test_size=test_size,
cv_folds=cv_folds)
        ml_dir = output_dir / "ml"
        ml_dir.mkdir(parents=True, exist_ok=True)

        # Save CSV files with new naming convention
        metric_df.to_csv(ml_dir / "vitamin D insufficiency (VDI)_model_metrics.csv",
index=False, encoding="utf-8-sig")
        imp_df.to_csv(ml_dir / "vitamin D insufficiency (VDI)_feature_importance.csv",
```

```
index=False, encoding="utf-8-sig")

        # Figure 1: Feature importance comparison (sorted by comprehensive importance descending)
        plot_ml_importance(imp_df, ml_dir / "vitamin D insufficiency (VDI)_feature_importance.tiff", no_show=no_show)

        # Figure 2: Stacked normalized importance (sorted by comprehensive importance descending)
        plot_normalized_importance(imp_df, ml_dir / "vitamin D insufficiency (VDI)_normalized_importance.tiff", no_show=no_show)

        shap_status = run_shap_analysis(data=data, seed=seed, test_size=test_size, ml_dir=ml_dir, no_show=no_show)

        print("\n" + "="*50)
        print("MACHINE LEARNING ANALYSIS RESULTS")
        print("="*50)
        print("\n[Model Performance Metrics]")
        print(pretty(metric_df))
        print("\n[Feature Importance (%)]")
        print(pretty(imp_df))

        if (ml_dir / "vitamin D insufficiency (VDI)_shap_mean_abs.csv").exists():
            shap_df = pd.read_csv(ml_dir / "vitamin D insufficiency (VDI)_shap_mean_abs.csv")
            print("\n[SHAP Mean Absolute Contribution]")
            print(pretty(shap_df))

        print(f"\n[SHAP Analysis] {shap_status}")
        print(f"\nOutput Directory: {ml_dir}")
        print(f"\nGenerated Figures:")
        print(f"    - Feature importance comparison → {ml_dir / 'vitamin D insufficiency (VDI)_feature_importance.tiff'}")
        print(f"    - Stacked normalized importance → {ml_dir / 'vitamin D insufficiency (VDI)_normalized_importance.tiff'}")
        print(f"    - SHAP summary plot → {ml_dir / 'vitamin D insufficiency (VDI)_shap_summary.tiff'}")


    def main() -> None:
        args = build_parser().parse_args()
        path = detect_data_path(args.data)
        data = load_data(path)
```

```python
    check_required_columns(data, FEATURES + [TARGET])
    print(f"Data file: {path}")
    print(f"Sample size: {len(data)}")
    print(f"Output directory: {args.output_dir}")
    run_ml_track(data, seed=args.seed, test_size=args.test_size, cv_folds=args.cv_folds, output_dir=args.output_dir, no_show=args.no_show)


if __name__ == "__main__":
    main()
```
