# Supplementary material for: A masking effect of vitamin D on ASD symptom severity: mediation by developmental level and altered predictive features in vitamin D insufficiency
Source: Front Psychiatry. 2026 Aug 28;17:1900932. doi: 10.3389/fpsyt.2026.1900932 (PMC13563125; doi:10.3389/fpsyt.2026.1900932)
Supplement: Supplementary file 3 [file DataSheet3.pdf]

Table 5. Path coefficients of the structural equation model for the low-age group

Variables	B	95% Confidence Interval		Z	P	β
		Lower	Upper			
**measurement model**						
adaptive behavior→developmental level	1.000	1.000	1.000			0.816
gross motor→developmental level	0.571	0.477	0.668	11.549	<0.001	0.537
fine motor→developmental level	0.918	0.805	1.041	15.085	<0.001	0.663
language→developmental level	0.849	0.699	1.076	9.116	<0.001	0.702
personal-social→developmental level	0.670	0.562	0.802	11.109	<0.001	0.620
**structural model**						
serum 25(OH)D_3 level→developmental level	0.103	0.047	0.154	3.768	<0.001	0.188
developmental level→CARS scores	-0.252	-0.326	-0.189	-7.062	<0.001	-0.416
serum 25(OH)D_3 level→CARS scores	0.027	0.003	0.048	2.372	0.018	0.080
**mediating effect**						
indirect effect	-0.026	-0.041	-0.012	-3.379	0.001	-0.078
direct effect	0.027	0.003	0.048	2.371	0.018	0.080
total effect	0.001	-0.025	0.025	0.051	0.959	0.002

Notes: B, unstandardized regression coefficient; β, standardized regression coefficient; 95% CI, 95% confidence interval for the unstandardized coefficient (B); z: z-value; *p*: *p*-value. Indirect effect = serum 25(OH)D_3 level → developmental level → CARS scores; direct effect = serum 25(OH)D_3 level → CARS scores; total effect = indirect effect + direct effect. Bootstrap with 5000 resamples was used to calculate 95% confidence intervals. Age, height, and weight were included as control variables.

Table 6. Path coefficients of the structural equation model for the high-age group

Variables	B	95% Confidence Interval		Z	P	β
		Lower	Upper			
**measurement model**						
adaptive behavior→developmental level	1.000	1.000	1.000			0.827
gross motor→developmental level	0.687	0.488	0.909	6.657	＜0.001	0.574
fine motor→developmental level	1.120	0.962	1.300	13.098	＜0.001	0.822
language→developmental level	0.872	0.639	1.214	5.954	＜0.001	0.714
personal-social→developmental level	0.931	0.685	1.288	6.346	＜0.001	0.775
**structural model**						
serum 25(OH)D_3 level→developmental level	0.129	0.037	0.220	2.730	0.006	0.224
developmental level→CARS scores	-0.324	-0.469	-0.204	-4.795	＜0.001	-0.429
serum 25(OH)D_3 level→CARS scores	0.038	-0.019	0.109	1.175	0.240	0.088
**mediating effect**						
indirect effect	-0.042	-0.076	-0.012	-2.495	0.013	-0.096
direct effect	0.038	-0.019	0.109	1.175	0.024	0.088
total effect	-0.003	-0.062	0.068	-0.101	0.919	-0.008

Notes: B, unstandardized regression coefficient; β, standardized regression coefficient; 95% CI, 95% confidence interval for the unstandardized coefficient (B); z: z-value; *p*: *p*-value. Indirect effect = serum 25(OH)D_3 level → developmental level → CARS scores; direct effect = serum 25(OH)D_3 level → CARS scores; total effect = indirect effect + direct effect. Bootstrap with 5000 resamples was used to calculate 95% confidence intervals. Age, height, and weight were included as control variables.

low-age group

```
# Load required R packages
library(tidyverse)          # Data manipulation and visualization
library(lavaan)              # Structural equation modeling
library(semPlot)             # Plotting SEM paths
library(readxl)              # Reading Excel files

# Read data file (please ensure the file path is correct)
data <- read_excel('C:/age at first diagnosis/vitamin/25(OH)D/Research data/total sample -low-age group.xlsx')

# Check data - confirm variables exist and have correct format
str(data)          # View data structure
head(data)         # View first few rows

# Construct structural equation model (Age removed, only D, Height, Weight as covariates)
model <- '
    # Measurement model - fix first loading to 1
    Developmental =~ 1*Adaptive + Gross_Motor + Fine_Motor + Language + Social_Skills

    # Structural model: Developmental and CARS regressed on D, Height, Weight (Age excluded)
    Developmental ~ vd1*D + h1*Height + w1*Weight
    CARS ~ b1*Developmental + cvd*D + h2*Height + w2*Weight

    # Covariances among all exogenous variables (Age removed)
    D ~~ Height + Weight
```

```
    Height ~~ Weight

    # Define indirect and total effects for D (your main interest)
    indirect_d_dev := vd1 * b1          # D -> Developmental -> CARS
    direct_d := cvd                     # D -> CARS direct
    total_d := cvd + indirect_d_dev     # total effect of D

    # Indirect effects for Height and Weight (optional)
    indirect_height_dev := h1 * b1
    indirect_weight_dev := w1 * b1

    # Residual correlations (based on theory or modification indices)
    Adaptive ~~ Fine_Motor
    Gross_Motor ~~ Fine_Motor
    Language ~~ Social_Skills
    Gross_Motor ~~ Social_Skills
    Fine_Motor ~~ Social_Skills
    Gross_Motor ~~ Height      # Added based on modification index (MI = 12.010)
    Language ~~ Height         # Added based on modification index (MI = 13.075); theoretically more plausible
'

# Fit model with RAM representation and bootstrap
fit <- sem(model,
           data = data,
           estimator = "ML",
           missing = "ML",                # Full information maximum likelihood for missing
```

```
            representation = "RAM",        # RAM representation to handle residual correlations
            se = "bootstrap",
            bootstrap = 1000)              # Increase to 500 or 1000 for stable SE

# View summary with fit measures, standardized coefficients, CIs
summary(fit,
        standardized = TRUE,
        fit.measures = TRUE,
        rsquare = TRUE,
        ci = TRUE)

# Check modification indices (optional, for potential improvements)
modindices(fit, sort = TRUE, minimum.value = 10)

# Plot path diagram (standardized coefficients, Age removed from labels)
semPaths(fit,
         what = "std",
         layout = "tree",
         fade = FALSE,
         residuals = TRUE,
         nCharNodes = 0,
         edge.label.cex = 0.9,
         sizeMan = 8,
         sizeLat = 10,
         edge.color = "black",
         nodeLabels = c("D", "Height", "Weight",
```

```
                    "Adaptive", "Gross_Motor", "Fine_Motor",
                    "Language", "Social_Skills", "CARS",
                    "Developmental"),
label.cex = 1.1,
curvePivot = TRUE)
```

high-age group

```r
# Load required R packages
library(tidyverse)          # Data manipulation and visualization
library(lavaan)             # Structural equation modeling
library(semPlot)            # Plotting SEM paths
library(readxl)             # Reading Excel files

# Read data file (please ensure the file path is correct)
data <- read_excel('C:/age at first diagnosis/vitamin/25(OH)D/Research data/total sample-high-age group.xlsx')

# Check data - confirm variables exist and have correct format
str(data)          # View data structure
head(data)         # View first few rows

# Construct structural equation model (Age removed, only D, Height, Weight as covariates)
model <- '
    # Measurement model - fix first loading to 1
    Developmental =~ 1*Adaptive + Gross_Motor + Fine_Motor + Language + Social_Skills

    # Structural model: Developmental and CARS regressed on D, Height, Weight (Age excluded)
    Developmental ~ vd1*D + h1*Height + w1*Weight
    CARS ~ b1*Developmental + cvd*D + h2*Height + w2*Weight

    # Covariances among all exogenous variables (Age removed)
    D ~~ Height + Weight
```

```
    Height ~~ Weight

    # Define indirect and total effects for D (your main interest)
    indirect_d_dev := vd1 * b1          # D -> Developmental -> CARS
    direct_d := cvd                     # D -> CARS direct
    total_d := cvd + indirect_d_dev     # total effect of D

    # Indirect effects for Height and Weight (optional)
    indirect_height_dev := h1 * b1
    indirect_weight_dev := w1 * b1

    # Residual correlations (based on theory or modification indices)
    Adaptive ~~ Fine_Motor
    Gross_Motor ~~ Fine_Motor
    Language ~~ Social_Skills
    Gross_Motor ~~ Social_Skills
    Fine_Motor ~~ Social_Skills
    Gross_Motor ~~ Height     # Added based on modification index (MI = 12.010)
    Language ~~ Height        # Added based on modification index (MI = 13.075); theoretically more plausible
'

# Fit model with RAM representation and bootstrap
fit <- sem(model,
           data = data,
           estimator = "ML",
           missing = "ML",             # Full information maximum likelihood for missing
```

```
          representation = "RAM",               # RAM representation to handle residual correlations
          se = "bootstrap",
          bootstrap = 1000)                      # Increase to 500 or 1000 for stable SE

# View summary with fit measures, standardized coefficients, CIs
summary(fit,
        standardized = TRUE,
        fit.measures = TRUE,
        rsquare = TRUE,
        ci = TRUE)

# Check modification indices (optional, for potential improvements)
modindices(fit, sort = TRUE, minimum.value = 10)

# Plot path diagram (standardized coefficients, Age removed from labels)
semPaths(fit,
         what = "std",
         layout = "tree",
         fade = FALSE,
         residuals = TRUE,
         nCharNodes = 0,
         edge.label.cex = 0.9,
         sizeMan = 8,
         sizeLat = 10,
         edge.color = "black",
         nodeLabels = c("D", "Height", "Weight",
```

```
                    "Adaptive", "Gross_Motor", "Fine_Motor",
                    "Language", "Social_Skills", "CARS",
                    "Developmental"),
label.cex = 1.1,
curvePivot = TRUE)
```
